# The Efficacy and Safety of Biologic Drugs in the Treatment of Moderate–Severe Crohn’s Disease: A Systematic Review

**DOI:** 10.3390/ph16111581

**Published:** 2023-11-08

**Authors:** Ana Avedillo-Salas, Sara Corral-Cativiela, Ana Fanlo-Villacampa, Jorge Vicente-Romero

**Affiliations:** Department of Pharmacology, Physiology and Legal and Forensic Medicine, Faculty of Medicine, University of Zaragoza, ES-50009 Zaragoza, Spain

**Keywords:** Crohn’s disease, biologic drugs, upadacitinib, vedolizumab, adalimumab, guselkumab, mirikizumab, ustekinumab, risankizumab, PF-00547659

## Abstract

Conventional therapy is the most commonly used treatment for Crohn’s disease (CD), but it does not always achieve disease control, which is why the use of biologic drugs is increasing. The aim of this study was to analyze the efficacy and safety of biologic drugs in adult patients diagnosed with moderate–severe CD. An intensive search was performed in PubMed, Web of Science and Medline to collect phase 2 or 3 clinical trials published between 2018 and 2023 that were randomized, placebo-controlled and double-blind trials analyzing the efficacy and safety of biologic drugs in adult patients diagnosed with CD. This systematic review was conducted according to the PRISMA statement. Thirteen clinical trials evaluating eight biologic drugs were included. Upadacitinib, vedolizumab, adalimumab, guselkumab, mirikizumab, ustekinumab and risankizumab showed statistically significant efficacy across different clinical, endoscopic, histological, genetic, biomarker or quality-of-life parameters. However, PF-00547659 only showed statistically significant results for the CDAI-70 at week 12. In terms of safety, the incidence and severity of adverse effects were analyzed, with all drugs being well tolerated and presenting a good safety profile since most adverse effects were mild. Biologic drugs can be considered an effective and safe option for the treatment of moderate–severe CD in adult patients with an inadequate response or intolerance to conventional therapy.

## 1. Introduction

Crohn’s disease (CD), together with ulcerative colitis (UC) and indeterminate colitis (IC), is a form of inflammatory bowel disease (IBD). CD presents with transmural and segmental inflammatory involvement, alternating between healthy and diseased bowel segments. It can affect any segment of the gastrointestinal tract, but the terminal ileum is the most common site, affecting up to 70% of patients [1,2,3,4,5].

Pathological inflammation of the intestinal tissue is mediated by an abnormal mucosal immune response to the intestinal bacterial flora. Despite much progress in research, the exact etiology is not known, but it is thought to be due to the influence of certain environmental factors (smoking, the environment or certain drugs, such as non-steroidal anti-inflammatory drugs or antibiotics) in genetically predisposed individuals [6,7,8,9].

### 1.1. Epidemiology

The incidence of IBD varies widely between countries. Although the prevalence of CD is higher in high-income countries, exceeding 0.3% in North America, Oceania and many countries in Europe, cases have increased in Asia, South America and the Middle East. Incidence is increasing worldwide, particularly in the western part of the world, coinciding with population growth and lifestyle changes brought about by economic and industrial development. However, cases are also occurring in developing countries that were previously largely unaffected by the disease, such as Brazil (with an annual percentage increase of +11.1%) and Taiwan (with an annual percentage increase of +4.0%). [1,2,10,11].

CD can begin at any age but has a peak incidence between the ages of 15 and 25 and is, therefore, more commonly diagnosed in young people. However, no significant gender differences have been found [1,3,4].

### 1.2. Diagnosis and Evolution

The diagnosis of Crohn’s disease requires a clinical history alongside complementary tests, which include stool and blood tests. The preferred method for complementary testing is ileocolonoscopy, with biopsies taken from the terminal ileum and the colonic segments. Additionally, 15% of patients have penetrating lesions at the point of diagnosis. Due to this, radiology techniques such as MRI (Magnetic Resonance Imaging), CT (Computed Tomography) and transabdominal ultrasound are deemed necessary to determine the scope and location of involvement. Recurrence of CD necessitates frequent imaging techniques for proper evaluation [1,3,4,12].

CD exhibits various phenotypic behaviors; however, luminal stenosis, perforation, gastrointestinal bleeding, abscess or fistula are common complications that most patients eventually encounter. The likelihood of developing complications increases as inflammation advances, affecting 19–36% of CD patients. Therefore, it can be deduced that within 5 years of diagnosis of patients, about 50% exhibit complications, while almost 70% develop complications within 10 years of diagnosis [5,13].

### 1.3. Therapeutic Possibilities

The main aim of treatment is to control inflammation, which occurs in flares with intermittent periods of remission, requiring treatment of the flares to reduce acute inflammation and maintenance treatment to reduce relapses. In some cases, however, the disease cannot be controlled, and two-thirds of patients require surgical treatment at least once during the course of the disease. As a result, treatment options have expanded quickly in recent years [1,3,12,14,15].

The management plan for a patient with CD must take into account several aspects, including disease activity, location and behavior (inflammatory, stenosing or fistulising), always taking into account the patient’s opinion. In addition, several factors influence the choice of the most appropriate medication for each case: previous response to treatment (especially when considering treatment of relapse, corticoid dependence or corticosteroid resistance) and the presence of extraintestinal complications [16].

The main pharmacological groups used in the treatment of CD are aminosalicylates (mesalazine), corticosteroids (budesonide, beclomethasone dipropionate and prednisone), immunosuppressants (methotrexate and thiopurines) and biologic drugs.

Aminosalicylates are a pharmacological group whose molecular structure includes mesalazine and has anti-inflammatory effects. They are mainly used in patients with mild CD with colonic involvement, but their efficacy and indication are currently questionable [12,17].

Corticosteroids are a group of drugs with anti-inflammatory and immunosuppressive activity indicated for the induction of CD remission in patients with moderate-to-severe inflammatory bowel disease or a mild flare refractory to mesalazine at appropriate doses. However, they are not used for maintenance treatment due to their lack of long-term efficacy and side effects (Cushing’s syndrome, hypertension, acne, osteoporosis, emotional lability, increased risk of infection, diabetes mellitus, insomnia, glaucoma and proximal myopathy). Although various mechanisms of action have been described, inhibition of pro-inflammatory proteins, decreased expression of pro-inflammatory cytokines, inhibition of T and B lymphocyte proliferation and promotion of a tolerogenic macrophage profile stand out [12,18].

Thiopurines are purine antagonists with immunomodulatory and cytostatic effects indicated for the treatment of autoimmune diseases, including CD. They are indicated in situations of corticosteroid dependence or resistance, treatment of perianal disease, maintenance of remission after a severe flare, co-treatment with some biologic drugs and prevention of post-operative relapse. Side effects are both idiosyncratic, including gastrointestinal intolerance, flu-like syndrome and pancreatitis, and dose-related, including myelotoxicity and hepatotoxicity [19,20,21].

Finally, methotrexate is an antimetabolite that interferes with the metabolism of folic acid. It competitively inhibits the conversion of dihydrofolate to tetrahydrofolate, which is essential for the synthesis of thymidine and purines. Its main indications are maintenance therapy in corticoid-dependent CD or prevention of immunogenicity associated with treatment with some biologic drugs such as anti-tumor necrosis factor (anti-TNF) antibodies. The main side effects include teratogenicity, gastrointestinal intolerance, myelotoxicity, hepatotoxicity and the risk of pulmonary fibrosis, in addition to those resulting from immunosuppression [12,22,23].

The first biologic drugs that were recommended were anti-TNF antibodies. These drugs are essential because they block tumor necrosis factor-alpha, a pro-inflammatory cytokine involved in several autoimmune diseases, including CD. This group of drugs includes infliximab, adalimumab, certolizumab pegol and golimumab, although only the first two are approved in Europe. Even though they are regarded as benign medications, they have a few negative results, like infusion reactions or hypersensitivity reactions. There are additional disadvantages to consider, as a noteworthy proportion of patients treated do not react positively to induction therapy (primary anti-TNF failure) or gradually lose their response (secondary failure) over time. Treatment options such as dose escalation or intensification, switching to another anti-TNF agent or changing the therapeutic target have been implemented to achieve therapeutic effectiveness in patients experiencing loss of response to a particular anti-TNF agent. However, their efficacy is not yet entirely comprehended [12,24].

**Figure 1:**
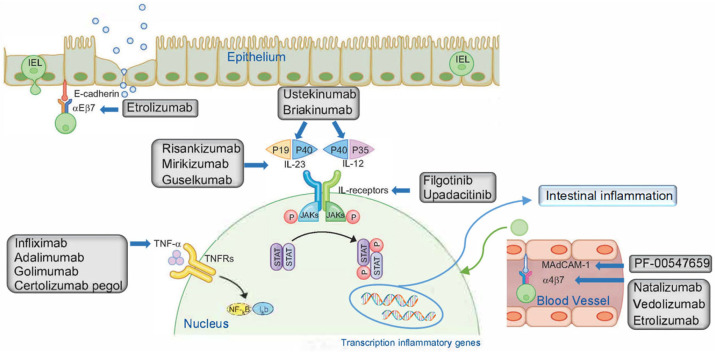
Therapeutic targets of biologic drugs for the treatment of inflammatory bowel disease. IEL, intraepithelial lymphocyte; Iκb, inhibitor of κB; IL, interleukin; JAK, Janus kinase; MAdCAM-1, mucosal vascular addressin cell adhesion molecule-1; NF-κB, nuclear factor-κB; P, phosphorylation; STAT, signal transducer and activator of transcription; TNF-α, tumor necrosis factor-α; TNFR, TNF receptor. Adapted from Na, S.Y. and Moon, W. [34].

Although until recently, anti-TNF agents were the only biologic drugs available, new classes of drugs have been developed in recent years (Figure 1):

- Janus kinase (JAK) inhibitors: upadacitinib and filgotinib. These are selective inhibitors of JAK1, which mediates IL-6 and IL-10 signaling. IL-6 is pro-inflammatory, unlike IL-10, which is anti-inflammatory. Therefore, inhibiting JAK1 may shift the balance, resulting in more or less inflammation [25];
- IL-12/23p40 inhibitors: ustekinumab and briakinumab. These are monoclonal antibodies directed against the p40 subunit shared by IL-12 and IL-23. The most common adverse effects are nasopharyngitis, respiratory infections and headache [12,15,25,26];
- Integrin inhibitors α4: natalizumab. This is a recombinant human monoclonal anti-integrin immunoglobulin antibody α4. The α4 subunit of the integrins α4β1 and α4β7 expressed by leukocytes binds to vascular cell adhesion molecule 1 (VCAM-1) and MAdCAM-1. It blocks leukocyte adhesion and migration from blood vessels into inflamed tissue [27,28];
- Integrin inhibitor α4β7: Vedolizumab is a humanized monoclonal antibody directed against the intestinal integrin α4β7, which is expressed on T and natural killer (NK) cells and immune cell subsets. The binding of this integrin to MAdCAM-1 allows lymphocytes to enter inflamed tissue. Although it selectively prevents the migration of immune system cells from the circulation to the mucosa, it also has side effects, including nasopharyngitis, headache, nausea, arthralgia, cough and abdominal pain [12,25,26,27,29,30,31];
- IL-23 inhibitors: risankizumab, mirikizumab and guselkumab. These are monoclonal antibodies that bind with high affinity to the p19 subunit of IL-23, which induces pro-inflammatory activity that activates T helper 17 cells [32,33];
- Anti-mucosal vascular targeting cell adhesion molecule-1 (MAdCAM-1) monoclonal antibody: PF-00547659. This is a fully human monoclonal antibody IgG2κ which, by binding to MAdCAM-1, prevents the entry of immune system cells into the inflamed intestinal mucosa [25,27];
- Inhibitors of the β7 subunit of the α4β7 and αEβ7 integrins: etrolizumab. This is a humanized monoclonal antibody that binds to the β7 subunit, thereby blocking the binding of the α4β7 integrin to MAdCAM-1 and the αEβ7 integrin to E-cadherin, a glycoprotein expressed mainly in epithelial cells [25,27].

Patients receiving maintenance immunosuppressive therapy for a moderate flare undergo treatment with biologic agents for both remission induction and maintenance therapy. In cases of severe flare-ups, intravenous corticosteroids are the preferred treatment, and hospitalization is necessary. If there is no response, biologic drugs will be implemented. In the event the patient had been on immunosuppressive medication before the severe flare-up, biologic drugs will serve as the preferred maintenance drugs. Similarly, in cases where patients are not receiving immunosuppressants but display signs of severity due to extension, severe onset, fistula pattern or perianal disease, treatment with biologic drugs may commence directly without prior immunosuppression [12].

### 1.4. Justification and Aim

Classical treatment is currently the most widely used therapeutic method for CD. However, it is noted that for certain individuals, symptoms cannot be managed by classical treatment, and as a result, the use of biologic therapies is increasing. Biological therapies have been a revolutionary advance in the treatment of moderate–severe CD in both the induction and maintenance phases, alleviating symptoms, promoting mucosal healing and reducing the need for surgery and hospitalization in patients. In addition, their use avoids the need for corticosteroid treatment, thereby improving patients’ quality of life. The use of these drugs can also make a significant contribution to the treatment of post-surgical relapses, avoiding the need for new surgery and improving patients’ quality of life, as this disease affects social relationships and is associated with a high incidence of absenteeism from work and school.

This review aims to summarize the evidence on the efficacy and safety of biologic drugs compared to placebo or other treatments in adult patients diagnosed with moderate-to-severe CD from clinical trials published between 2018 and 2023.

## 2. Materials and Methods

### 2.1. Search Strategy

The search was conducted using PubMed, Web of Science and Medline. Studies were identified by combining the name “Crohn’s disease” with the keywords infliximab, adalimumab, certolizumab pegol, golimumab, tofacitinib, upadacitinib, ustekinumab, briakinumab, natalizumab, vedolizumab, risankizumab, mirikizumab, filgotinib, baricitinib, decernotinib, guselkumab, PF-00547659, and etrolizumab and their drug class. MeSH (Medical Subject Headings) terms and the Boolean operators “AND” and “OR” were also used in the search.

**Figure 2:**
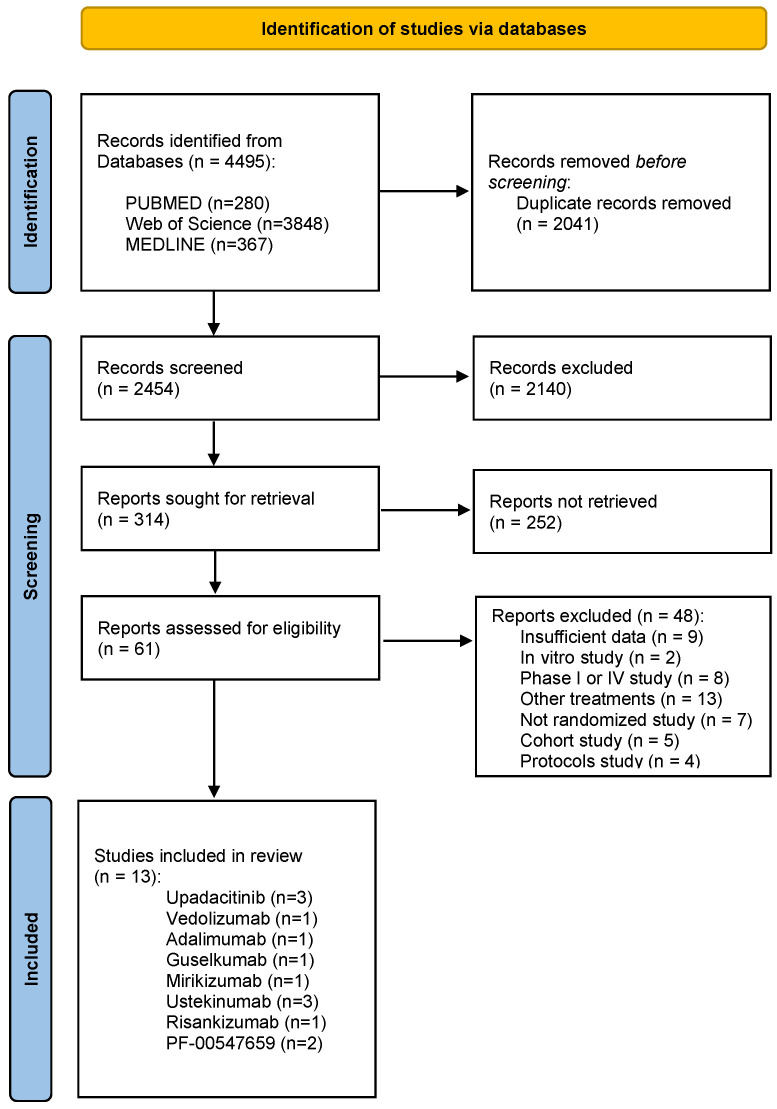
PRISMA 2020 flow diagram illustrating the study selection process [35].

This study was conducted according to the PRISMA statement [35]. Articles published between 2018 and 2023 were retrieved. After removing duplicates, titles and abstracts were screened, excluding those that did not meet the inclusion criteria. The remaining records were then assessed for eligibility by careful review of their full texts. A flowchart illustrating the study selection process is shown in Figure 2.

### 2.2. Inclusion Criteria

Regarding the types of studies, double- or triple-blind randomized clinical trials in phases 2 or 3 were included. The other inclusion criteria were proposed according to the PICO algorithm (Table 1).

### 2.3. Exclusion Criteria

The proposed exclusion criteria for this systematic review were: (a) studies with insufficient data; (b) in vitro, in silico and in vivo animal studies; (c) commentaries, expert opinions, case reports, letters to the editor, reviews, protocols and study registries, observational studies, systematic reviews and meta-analyses; (d) phase 1 clinical trials; (e) studies that do not include as an intervention at least one of the biologic drugs evaluated in this systematic review; (f) studies with medicinal plants; and (g) studies that include pregnant or lactating women.

### 2.4. Data Collection and Analysis

Data gathering should be conducted by three reviewers independently (A.A.-S., A.F.-V. and J.V.-R). The following information was extracted from each of the included trials: (a) clinical trial registration number, (b) author, (c) publication date, (d) trial design, (e) participant characteristics, (f) interventions delivered with each dose regimen, (g) comparison group dose regimen, (h) age of participants, and (i) outcomes. The quality of evidence was assessed following the Grading of Recommendations Assessment, Development and Evaluation (GRADE) system [36,37].

### 2.5. Outcomes and Definitions

The effectiveness and safety of biologic drugs used in the treatment of CD, compared to placebo or other drugs, have been assessed by analyzing the outcomes presented in Table 1, considering the following criteria:

- Clinical:
  - SF and AP: an increase in their score indicates a worsening of the patient’s clinical condition [38];
  - CDAI: assess the activity of DC based on clinical criteria, whereby <150 points indicate remission, 150–250 points indicate mild flare-ups, 250–350 points indicate moderate flare-ups and >350 points indicate severe flare-ups. A decrease of ≥70 points or ≥25% of the previous score indicates a clinical response [38];
  - PRO: a questionnaire derived from the CDAI that determines the severity of CD by adding up the weighted scores of the SF and AP. An increase in the score indicates a worsening of the patient’s symptoms [38].
- Endoscopy:
  - SES-CD: assess the activity of CD based on endoscopic criteria. A score of 0–2 indicates inactive disease, 3–6 indicates mild activity, 7–15 represents moderate activity, and >15 points represents severe activity [39];
  - CDEIS: assess the severity of CD based on endoscopic criteria. A score of 0–2 indicates inactive CD, 3–9 points: mild activity, 9–12 points: moderate, and >12 points: severe [40].
- Biomarkers:
  - FCP, CRP, hs-CRP and lactoferrin: these are biomarkers that express inflammation;
  - MAdCAM: an increase in serum concentrations is indicative of increased inflammation in the intestinal mucosa.
- Quality of life:
  - IBDQ: The minimum score is 32, and the maximum is 224. The interpretation of the obtained score is as follows: 32–95 points indicate low quality of life, 96–159 points indicate moderate quality of life, and 160–224 points indicate high quality of life [41].
  - EQ-D5 VAS: indicate worse quality of life with higher scores. A decrease in the score indicates an improvement in quality of life [42].
  - WPAI: evaluate how a patient’s general health status affects their daily activities and work productivity. A decrease in the score indicates an improvement in quality of life [43].
- Histology:
  - GHAS: determine the activity of CD based on histological criteria. A higher score denotes a higher activity of CD.

## 3. Results

### 3.1. Upadacitinib

Upadacitinib was analyzed in this review, comprising three studies conducted within the CELEST clinical trial. This trial lasted 52 weeks and was conducted across multiple centers. This study was randomized, parallel, double-blind and placebo-controlled. The participants in the trial were between 18 and 75 years old and had been diagnosed with moderate CD for a minimum of three months. Patients with ileal, colic or ileocolic CD with a CDAI of 220–450, FS ≥ 2.5 and an AP score ≥ 2, as well as evidence of intestinal mucosal inflammation as determined by SES-CD. Patients who were unresponsive or had an intolerance to azathioprine, mercaptopurine or methotrexate, as well as those receiving stable doses of aminosalicylates, oral corticosteroids, antibiotics or anti-TNF, were also included. Azathioprine and mercaptopurine treatment ceased at least 10 days before the commencement of the study, and corticosteroids were gradually tapered from the second week until discontinuation.

The phase 2 clinical trial conducted by Sandborn WJ et al. [44] involved the randomization (1:1:1:1) of patients to receive 16 weeks of oral induction therapy. A placebo group (*n* = 37) and four upadacitinib treatment groups at doses of 3 mg/12 h (*n* = 39), 6 mg/12 h (*n* = 37), 12 mg/12 h (*n* = 36), 24 mg/12 h (*n* = 36) and 24 mg/24 h (*n* = 35) were established. Participants who successfully completed the previous treatment were re-randomized (1:1:1) to undergo maintenance treatment with the aforementioned oral medication. The doses administered were 3 mg/12 h (*n* = 61), 6 mg/12 h (*n* = 23), 12 mg/12 h (*n* = 59) and 24 mg/24 h (*n* = 37), all of which were given for a duration of 36 weeks.

Clinical remission efficacy at week 16 was assessed using two criteria: clinical remission 1.5/1.0 (mean daily frequency of loose/very soft stools (SF) ≤ 1.5 and daily abdominal pain (AP) score ≤ 1) and clinical remission 2.8/1.0 (SF ≤ 2.8 and AP score ≤ 1, in patients with PS ≥ 4 or AP ≥ 2).

For the first criterion, the cohort administered 6 mg/12 h showed the highest remission rate at 27% (*p* < 0.1), in contrast to the placebo group’s rate of 14%. As for the subsequent measure, the most effective dosage for remission was 24 mg/12 h at 37% (*p* < 0.05), compared to the placebo cohort’s rate of 12%.

Furthermore, this study revealed that clinical remission 1.5/1.0 was attained by 33% (*p* < 0.05) of patients who received 24 mg/12 h upadacitinib and were not undergoing corticosteroid therapy at baseline, as opposed to 0% in the placebo group.

Moreover, upadacitinib administration resulted in clinical remission (CDAI < 150) in 20–39% of treated individuals, compared to 16% of placebo-treated ones. However, an analysis focused solely on patients treated with corticosteroids at baseline indicates that clinical remission was achieved in 41% and 33% of patients receiving the 12 mg and 24 mg doses twice daily, respectively (*p* < 0.05), in comparison to 0% in the placebo group.

There was a decrease in inflammation of the intestinal lining, indicated by a significant reduction in hs-CRP levels among all patients treated with upadacitinib. Particularly noteworthy were the patients receiving 6 and 24 mg/12 h doses, who experienced mean reductions of 4.6 and 3.2 mg/dL, respectively (*p* < 0.05). The only exception was those receiving the 3 mg/12 h dose and the placebo group, which reported mean reductions of 0 mg/dL.

The effectiveness was evaluated at week 52 by categorizing patients into two groups: responders, who achieved a clinical response at week 16, and double responders, who attained a clinical and endoscopic response at week 12 and 16, respectively. The findings indicate that participants who received the 12 mg/12 h dose, including responders and double responders, had a greater proportion of patients achieving clinical remission 2.8/1.0 (73% and 52%, respectively), as well as endoscopic response 50% (69% and 45%, respectively) and CDAI < 150 (69% and 55%, respectively), when compared with the control group who received the 3 mg/12 h dose. While the 6 mg/12 h dosage demonstrated the highest percentage of patients achieving a CDAI < 150 without prior steroid treatment, the responder group and double responder group had 100% and 67% success rates, respectively, compared to 50% and 47% in the control group.

In terms of safety, the most common adverse events were headache, worsening CD, abdominal pain, fatigue, upper respiratory tract infections, urinary tract infections, nausea, vomiting and acne. Over the course of 16 weeks, the 3 mg/12 h group demonstrated the highest percentage of adverse events (87.2%), with the 6 mg/12 h group reporting the lowest (78.4%) in comparison to 73% in the placebo group. Notably, the group receiving a dose of 12 mg/12 h exhibited the greatest percentage of grave adverse events (27.8%), whereas the groups receiving a dose of placebo and 6 mg/12 h both had the lowest percentages (5.4% for both). As for discontinuation induced by adverse events, the group receiving 12 mg/12 h had the highest rate (25%), while the group receiving 6 mg/12 h exhibited the lowest rate (2.7%).

At week 52, data were analyzed, and it was found that the proportion of adverse events was highest in patients receiving upadacitinib at a dosage of 3 mg/12 h (75%), while it was lowest at 6 mg/12 h (60.9%). The highest proportion of serious adverse events was also observed in patients receiving the 3 mg/12 h dose (25%), but the lowest incidence of such events occurred at the 12 mg/12 h dose (8.5%). Regarding discontinuation due to adverse events, the 12 mg/12 h dosage exhibited the highest incidence (8.5%), whereas the 6 mg/12 h dose had the lowest incidence (0%).

During the induction period (weeks 0–16), a total of nine serious infections occurred among patients receiving the study drug. Additionally, there was one outbreak of herpes zoster, one incidence of cutaneous melanoma in a patient taking azathioprine in the 24 mg/12 h group and one case of acute myocardial infarction leading to treatment discontinuation in a 67-year-old male smoker who was receiving upadacitinib 12 mg/12 h. Additionally, one case of acute myocardial infarction led to treatment discontinuation in a 67-year-old man who received upadacitinib 12 mg/12 h. The patient had a history of smoking, diabetes mellitus and a family history of myocardial infarction. Two cases of intestinal perforation were reported, which were associated with severe infection and required surgery. Both cases occurred in areas of the bowel that were actively inflamed and affected by Crohn’s disease. The case occurring on day 36 of treatment was on the 24 mg/24 h dose, while the case occurring on day 41 was on 24 mg/12 h. Technical abbreviations such as CD are always first explained. Additionally, one case of mesenteric venous thrombophlebitis was reported in a patient on the 3 mg/12 h dose; however, no cases of deep vein thrombosis or pulmonary embolism were observed.

Maintenance treatment between weeks 16 and 52 caused six cases of severe infections: five on the 3 mg/12 h dose and one on the 12 mg/12 h dose. Moreover, two patients under the 24 mg/12 h dose suffered from an outbreak of herpes zoster, and two cases of malignancy were also recorded. The initial case was Hodgkin’s lymphoma diagnosed in a 29-year-old male patient who was administered 6 mg/12 h for 16 weeks initially, followed by 12 mg/12 h for 36 weeks afterward. The patient had a family history of lymphoma and had previously undergone treatment with mercaptopurine and multiple biologic agents, including adalimumab, infliximab, vedolizumab and natalizumab. The second case involved a 62-year-old man who developed a malignant neoplasm of the thymus. He was administered 24 mg/24 h for the initial 16 weeks, followed by 12 mg/12 h for the following 13 weeks. He had no family history of cancer but was previously treated with mercaptopurine, azathioprine, methotrexate and biologic drugs such as infliximab and vedolizumab. One case of aspiration pneumonia misdiagnosed as myocardial infarction occurred in a 55-year-old man with a history of obesity, hypertension, diabetes mellitus, gout and gastro-oesophageal reflux who received the 3 mg/12 h dose.

The Peyrin-Biroulet L et al. [45] study was conducted during the phase 2b CELEST clinical trial. During the maintenance phase, patients were randomly allocated (1:1:1) to receive upadacitinib at doses of 3 mg/12 h (*n* = 32), 12 mg/12 h (*n* = 29) and 24 mg/24 h (*n* = 19). Following a protocol amendment, a new randomization (1:1:1) was implemented, with patients administered upadacitinib in dosages of 3 mg/12 h (*n* = 32), 6 mg/12 h (*n* = 14) and 12 mg/12 h (*n* = 29).

Their efficacy was evaluated during weeks 8, 16 and 52 using the IBDQ, EQ-D5 VAS and WPAI assessment tools.

The groups that exhibited the most prominent alteration in IBDQ score from its baseline during week 8 were those administered with 6 and 24 mg/12 h doses, with a satisfactory difference of 35 points (*p* ≤ 0.05) and 40 points (*p* ≤ 0.01) each, in comparison to the placebo group that witnessed a rise of only 17 points. Likewise, at week 16, it was again the 6 and 24 mg/12 h doses that showed the most significant surge in the score, counting 39 and 41 points individually (*p* ≤ 0.01 in both scenarios). In contrast, the placebo group only experienced a rise of 13 points. However, the 12 mg/12 h dose showed the greatest increase at week 52 (71 points) in comparison to those receiving the 3 mg/12 h dose (43 points).

In the examination of IBDQ remission (score ≥ 170, high quality of life) at week 8, a higher proportion of patients treated with upadacitinib achieved remission when compared to the placebo group. The patients who were given the 24 mg/12 h dose exhibited the highest percentage of remission at 36% (*p* ≤ 0.01). Similar trends were observed in week 16 with the 6, 12 and 24 mg/12 h doses, where the percentages of remission were 32%, 33% and 39%, respectively (*p* ≤ 0.05). Although the proportion of patients treated with upadacitinib who achieved remission was higher at week 52 than at week 16, this difference was not statistically significant compared to the control group receiving a 3 mg/12 h dose.

In regard to IBDQ response (increase in score ≥ 16 points from baseline), a greater proportion of patients treated with upadacitinib at doses of 6 and 12 mg twice daily (62% (*p* ≤ 0.05) and 58% (*p* ≤ 0.1), respectively), and 24 mg once daily [57% (*p* ≤ 0.1)], achieved an IBDQ response at week 8 compared to those who received placebo (38%). At week 16, the 6 and 24 mg every 12 h doses showed a similar increase in IBDQ (57% and 56%, respectively) as compared to placebo (24%) (*p* ≤ 0.01 in both cases). Similarly, at week 52, the 6 and 12 mg every 12 h doses exhibited an increase of 79% and 69%, respectively (*p* ≤ 0.05), compared to 44% in the 3 mg/12 h group.

The EQ-D5 VAS exhibited the most considerable changes at week 8 with the 6 and 24 mg/12 h doses (15- and 14-point increase (*p* ≤ 0.1), respectively) when compared to the placebo. The 6 mg/12 h dosage registered a 17-point increase (*p* ≤ 0.05) during week 16, whereas the 24 mg/12 h dose recorded a 15-point increase (*p* ≤ 0.05) when compared to the placebo. At week 52, the 12 mg/12 h group exhibited the largest score increase of 36 points; however, this increase was not statistically significant. Conversely, the 24 mg/24 h dose produced a statistically significant increase of 8 points (*p* ≤ 0.05) compared to the 3 mg/12 h dose.

Considering outcomes of the WPAI questionnaire, changes were deemed drug-related only if there was a minimum reduction of 7% in each aspect evaluated, including presenteeism, absenteeism, general work impairment (presenteeism and absenteeism) and activity impairment (disability). A larger percentage of patients who received upadacitinib at 6 and 24 mg/12 h dosages accomplished this objective when compared to the ones who were given a placebo (*p* ≤ 0.05 and 0.01 correspondingly). At week 52, the results remained elevated across all doses of the medication compared to week 16. However, there were no statistically significant enhancements observed in contrast to the 3 mg/12 h dose.

The efficacy and safety of the CELEST study were evaluated by Mohamed MEF et al. [46] using distinct parameters from that of prior studies. 

At week 12, clinical response (reduction ≥ 30% from baseline in SF and/or AP score, none worse than baseline) and clinical remission (2.8/1) were examined for efficacy. A value of 0 (SF ≤ 2.8 and AP score ≤ 1, in patients with SF ≥ 4 or AP ≥ 2) and a CDAI < 150 reduced inflammation by 25% (decrease ≥ 25% in SES-CD from baseline) and improved inflammation by 50% (decrease >50% in SES-CD) and were inflammation-free (SES-CD ≤ 4 points and a decrease ≥ 2 points from baseline). The 24 mg/12 h dose demonstrated the most significant increase in patient success rates (61%, 33%, 36%, 57%, 36%, 36% and 20%, respectively) across all parameters compared with the placebo group (35%, 12%, 21%, 15%, 3% and 0%, respectively). The success rates for the placebo group were 21%, 15%, 15%, 3% and 0%, respectively.

Regarding safety, the most frequent adverse reactions with all plasma concentrations until week 16 were herpes zoster virus infection, pneumonia and severe infections. By week 16, they were lymphopenia ≥ grade 1, 2 and 3 and hemoglobin decrease ≥ 2 g/dL, with 28.9% of patients with a Cave (34,100) having lymphopenia ≥ grade 1. However, there was no apparent increase in adverse reactions with continued elevation of plasma concentrations.

### 3.2. Vedolizumab

The VISIBLE2 study analyzed by Vermeire S et al. [47] was a phase 3, multicenter, randomized, parallel, double-blind, placebo-controlled trial lasting 52 weeks. Patients aged 18 to 80 years, diagnosed with moderate-to-severe Crohn’s disease (CD) at least three months prior and having an inadequate response or intolerance to corticosteroids, immunosuppressants and/or anti-TNF, were selected for the trial.

Prior to randomization, an open-label induction study was conducted in which 644 patients received 300 mg intravenous vedolizumab at weeks 0 and 2. At week 6, those who achieved a clinical response (≥70 points decrease in CDAI from baseline) were then randomly allocated (2:1) to receive subcutaneous vedolizumab 108 mg every 2 weeks from week 6 to week 50 and were evaluated at week 52 (*n* = 275). The control group received subcutaneous placebo, also every 2 weeks (*n* = 135).

At week 6, 50.6% and 84.4% of randomly assigned patients achieved clinical remission (CDAI ≤ 150) and clinical response (decrease in CDAI ≥ 70 points from baseline), respectively, demonstrating efficacy.

At week 52 of the study, drug-treated patients achieved clinical remission at a rate of 48%, significantly higher than placebo-treated patients who achieved remission at a rate of 34.3% (*p* = 0.008). Clinical response rates were 52% and 44.8%, respectively (*p* = 0.167).

The achievement of clinical remission at week 52 was analyzed in relation to previous treatment in patients. Among those previously treated with oral corticosteroids who tapered off during the study, a significantly higher percentage of vedolizumab-treated patients (45.3%) achieved remission compared to placebo-treated patients (18.2%) (*p* = 0.002). Explanations of technical term abbreviations were provided when first used. In patients who had not received prior anti-TNF treatment, remission was attained in 48.6% of those treated with vedolizumab and in 42.9% of those given a placebo (*p* = 0.591). On the other hand, in patients who had previously failed anti-TNF therapy, the remission rate was 46.4% for vedolizumab and 28.8% for placebo (*p* = 0.019). Remission was attained by 41% of the patients receiving the study medication and 18.2% of those receiving the placebo who had never been treated with corticosteroids or anti-TNF. For those who had previously experienced a non-response to anti-TNF treatment but not to corticosteroids, remission was observed by 46.2% and 15%, respectively.

Some biomarkers were analyzed, revealing that those treated with vedolizumab had a higher proportion (60.5%) of normal FCP concentrations (≤250 µg/g) in comparison to those treated with a placebo (31.7%), indicating a decrease in inflammation. At baseline, CRP was elevated (>5 mg/L) in 61.1% of participants in the study drug group and 59.7% in the placebo group. However, at week 52, 23.2% and 17.5%, respectively, had normalized to levels < 5 mg/L.

In regard to safety, at week 52, 73.5% of patients treated with vedolizumab experienced adverse events compared to 76.1% in the placebo group. Gastrointestinal issues, such as abdominal pain and worsening of CD, were the most frequent adverse events reported. Serious adverse events occurred in 8.4% of patients treated with the study drug compared to 10.4% in the placebo group. Treatment discontinuation due to adverse events occurred in a total of 22 patients, of whom 11 were treated with the study drug (4%), and the remaining 11 were in the placebo group (8.2%).

In particular, upper respiratory tract infections, nasopharyngitis and injection site reactions showed a higher occurrence in the study drug group compared to the placebo group. On the other hand, anaphylaxis or anaphylactic shock, infections and neoplasms were more prevalent in the placebo group. In addition, two cases of malignancy were reported in the vedolizumab group and three in the placebo group.

In relation to infection rates, the vedolizumab group had 86 cases, while the placebo group had 46 cases, all of which were deemed to be serious. These included eleven abdominal and gastrointestinal infections, seven of which were in the placebo group and one Clostridium difficile infection. No infections were deemed to be linked to the study drug, with the exception of one case of gastroenteritis, which resulted in all patients making a complete recovery. Nevertheless, two patients in the vedolizumab group had to discontinue treatment (one for an anal abscess, which was considered moderate in severity, and one for an abdominal abscess, which was considered severe).

Notably, there were no deaths in either the drug or placebo groups.

### 3.3. Adalimumab

The SERENE trial reviewed by D’Haens GR et al. [48] was a phase 3, multicenter, randomized, parallel, double-blind, placebo-controlled trial lasting 56 weeks. Eligible patients were aged 18–75 years, diagnosed with active moderate-to-severe CD (CDAI 220–450) despite prior treatment with standard therapy, and had endoscopic evidence of mucosal inflammation (SES-CD ≥ 6 or in the case of exclusive ileal disease ≥ 4, excluding stenosing component). Patients who were unresponsive to or intolerant of infliximab were also included in the trial.

During the induction phase, the study enrolled patients through a randomization process (3:2), assigning them to either the high induction regimen (HIR) (*n* = 308) or the regular induction regimen (SIR) (*n* = 206). Patients with HIR received adalimumab 160 mg at weeks 0, 1, 2 and 3 and 40 mg from week 4 to week 12. Patients with SIR received adalimumab 160 mg at week 0, placebo at week 1, adalimumab 80 mg at week 2, placebo at week 3 and adalimumab 40 mg from week 4 to week 12. All patients who completed the induction study were re-randomized (1:1) to two treatment strategies: clinically adjusted (CA) (*n* = 92) and therapeutic drug monitoring (TDM) (*n* = 92). CA consisted of increasing the dose of adalimumab by 40 mg every week if the CDAI score was ≥220 or the hs-CRP level was ≥10 mg/L (the most common cause of dose increase). The TDM strategy consisted of 40 mg adalimumab every week if the concentration was <5 µg/mL (the most frequent cause of dose escalation), 40 mg every other week if it was >10 µg/mL and 40 mg every week if it was 5–10 µg/mL, CDAI score ≥ 220 or hs-CRP level ≥ 10 mg/L. The maintenance phase started at week 12 and ended at week 56.

Efficacy at week 4 was analyzed by clinical remission (CDAI < 150), which was achieved by 43.5% of patients treated with HIR and 43.7% of patients treated with SIR (*p* = 0.939).

At week 12, 42.9% of patients in the HIR group and 39.3% of patients in the SIR group achieved an endoscopic response (decrease ≥ 50% in SES-CD from baseline or decrease ≥ 2 points if SES-CD = 4 at baseline) (*p* = 0.462).

Clinical remission was achieved by 62.3% of patients in the HIR group compared to 51.5% in the SIR group (*p* = 0.008), and clinical response (reduction in CDAI score ≥ 70%) occurred in 83.4% of patients in the HIR group compared to 74.8% in the SIR group (*p* = 0.015).

Efficacy at week 56 was assessed by clinical remission (*p* = 0.497), endoscopic response (*p* = 0.824), endoscopic remission (SES-CD ≤ 4 and reduction ≥2 points from baseline) (*p* = 0.621), clinical remission without corticosteroids (CDAI < 150 and discontinuation of corticosteroid therapy in patients taking corticosteroids before baseline) (*p* = 0.636) and complete remission (clinical and endoscopic remission) (*p* = 0.507). It was observed that a higher percentage of patients in the CA group (70.7%, 44.6%, 31.5%, 76.9% and 29.3%, respectively, for each of the above parameters) achieved the objectives compared to the TDM group (66.3%, 43.5%, 29.3%, 73.2% and 26.1%, respectively).

Over 70% of patients who achieved clinical remission at week 12 maintained it at week 56, with similar rates in the CA and TDM groups. Despite marginally higher rates being observed in the CA group as compared to the TDM group, the endoscopic response was sustained at week 56 in more than 50% of patients in both groups.

In terms of safety, during the induction period (weeks 0–12), it was observed that the rates of adverse events were similar in the HIR group (60.1%) compared to the SIR group (64.6%), of which 24.4% and 26.2%, respectively, were considered possibly related to the study drug. The most common adverse events in both groups were headache, worsening of CD, nasopharyngitis, arthralgia, nausea and dizziness.

From week 12 to week 56 of the maintenance period, the proportion of observed trends was similar between the CA group (70.6%) and the TDM group (69.7%). There were 26.6% and 30.3% reported incidents in the CA and TDM groups, correspondingly, which could potentially be associated with the study drug. The most common AEs were headache, exacerbation of CD, nasopharyngitis, arthralgia and diarrhea.

Most of the adverse events that occurred during the induction period were considered mild or moderate, and only 17 patients in the HIR group (5.5%) and 13 in the SIR group (6.3%) experienced severe adverse events. In the maintenance period, the rates were 6.4% in the CA group and 5.5% in the SIR group. With regard to infections, the percentage was similar in the HIR (22.4%) and SIR (23.8%) groups, all of which were considered mild, with the exception of two reports of severe infection in each group. The same trend was observed in the CA (33.9%) and TDM (34.9%) groups, all of which were considered mild, with the exception of three serious infections in the TDM group. Although no opportunistic infections occurred during the maintenance period, there were three cases in the induction period, one in the HIR group and two in the SIR group.

Additionally, at week 8, a single incident of papillary cell renal carcinoma was reported in the HIR group, yet this was unrelated to the medication in question. It is important to note that no occurrences of cancer were documented within the remaining three groups.

With regard to adverse events resulting in cessation of treatment, 4.2% of patients in the HIR group and 3.9% of patients in the SIR group discontinued treatment during the induction period, while 7.2% and 8.3% of patients in the CA and TDM groups discontinued treatment during the maintenance period. Notably, there were no deaths in any of the four groups.

This study’s comparison of the four groups has led to the conclusion that treatment-emergent adverse events and treatment-emergent serious adverse events took place more frequently in patients belonging to the CA group, as opposed to the other groups (70.6% and 6.4%, respectively). Conversely, the TDM group experienced a higher occurrence of serious adverse events, those leading to patient discontinuation, and treatment-emergent adverse events that could be related to the drug under study (6.4%, 8.3% and 30.3%, respectively).

### 3.4. Guselkumab

The GALAXI-1 trial, analyzed by Sandborn WJ et al. [49], was a phase 2, multicenter, randomized, parallel, double-blind, placebo-controlled trial of 12 weeks’ duration. The patients selected were over 18 years of age and had been diagnosed with active moderate-to-severe CD for at least 3 months. This diagnosis was based on clinically active CD (CDAI 220–450, SF > 3 or AP > 1) and endoscopic evidence of ileocolic CD (SES-CD ≥ 3 on screening endoscopy with a score for ulceration ≥ 1). The inclusion of patients with SES-CD 3 (ileal disease only) and SES-CD 3–6 (colic or ileocolic disease) was limited to a maximum of 10% of the study population. Additionally, patients who had not responded well to conventional treatment or had developed intolerance, as well as those who had received but not responded positively to biological therapy, were included.

Patients were randomized (1:1:1:1:1) to receive placebo (*n* = 61) at weeks 0, 4 and 8 (control group), ustekinumab 6 mg/kg intravenously at week 0 and 90 mg subcutaneously at week 8 (*n* = 63), guselkumab 200 mg (*n* = 61), 600 mg (*n* = 63) and 1200 mg (*n* = 61) at all three doses administered intravenously at weeks 0, 4 and 8. The ustekinumab arm was included as the reference arm because even though guselkumab was not compared to ustekinumab in this phase, it is a target for phase 3.

Efficacy at week 12, as assessed by the CDAI score at baseline compared to week 12, showed a mean decrease of 36.2 points with placebo. However, the decrease was much greater, measuring 160.4, 138.9 and 144.9 points with guselkumab 200, 600 and 1200 mg, respectively (*p* < 0.05 in all cases).

Clinical remission (CDAI < 150) (*p* = 0.05), clinical response (CDAI < 150 or reduction ≥ 100 points) (*p* = 0.05), PRO remission (unweighted CDAI component of AP ≤ 1 and unweighted CDAI component of mean SF ≤ 3) (*p* = 0.05), endoscopic remission (improvement ≥ 50% in baseline SES-CD score) (*p* = 0.05) and clinical biomarker response (clinical response and ≥ 50% reduction in baseline CRP or CPF) (*p* = 0.05). For all of these endpoints, the proportion of patients reaching the target was higher in the guselkumab group (35.7–65.9%) compared to the placebo group (6.6–24.6%).

The same trend was observed when analyzing only those patients who previously had an inadequate response or intolerance to biologic drugs, as clinical remission, clinical response, PRO remission and endoscopic response (improvement ≥ 50% in baseline SES-CD score or SES-CD ≤ 2) were achieved by a higher percentage of patients treated with the study drug (47.5%, 62.4%, 40.6% and 30.7%, respectively) compared to those receiving placebo (10%, 20%, 13.3% and 13.3%, respectively). When analyzing the same factors in patients who either showed intolerance or had an inadequate response to conventional treatment, a higher percentage of patients treated with guselkumab (59.5%, 70.2%, 45.2% and 41.7%, respectively) achieved their objective compared to the placebo group (22.6%, 29%, 19.4% and 9.7%, respectively).

Regarding safety at week 12, the placebo group had the highest incidence of adverse events at 60% compared to 45.7% in the guselkumab group. However, there was no clear association between the dosage of guselkumab and the proportion of patients who experienced adverse events.

The incidence of serious adverse events was 5.7% in the placebo group and 3.7% in the guselkumab group. Among the serious adverse events, toxic hepatitis was reported in a 44-year-old woman who received 1200 mg intravenous guselkumab at weeks 0, 4 and 8 and a single 200 mg subcutaneous dose at week 12. The patient’s liver parameters were considered normal at the beginning of the study and week 8 when symptoms first appeared. Liver tests at week 12 showed alanine aminotransferase and aspartate aminotransferase levels 15 and 10 times the upper limit of normal, respectively. However, bilirubin levels were normal, and alkaline phosphatase was slightly elevated. Although no clear cause was found, the patient recovered without sequelae, and her liver enzymes normalized within three months. The patient discontinued study treatment and was therefore excluded from the study.

Infections occurred in 21.4% of the placebo group and 15.1% of the study drug group. However, the percentage of patients with at least one serious infection was 0% in the placebo group and 1.4% in the guselkumab group. Specifically, there were three cases of serious infections reported in the guselkumab group: anal abscess at the 200 mg dosage, viral gastroenteritis and enterovesical fistula at the 600 mg dosage. Nevertheless, none of the serious infections were determined to be related to the drug.

There were no cases of hypersensitivity reactions, active tuberculosis, opportunistic infections or mortality.

### 3.5. Mirikizumab

The SERENITY trial reviewed by Sands BE et al. [50] was a phase 2, multicenter, randomized, parallel, double-blind, placebo-controlled, 52-week trial. Patients selected were aged 18–75 years and diagnosed with moderate–severe CD (FS ≥ 4, AP ≥ 2, SES-CD ≥ 7 in ileocolic CD or ≥ 4 in ileal CD) with a minimum duration of 3 months. To be eligible for the study, patients must have undergone previous treatment with aminosalicylates, mercaptopurine, azathioprine and corticosteroids in the presence of intolerance or non-response, corticosteroid dependency and/or at least one biologic (anti-TNF, vedolizumab or experimental, except those acting on IL-23) with or without a history of inadequate response or non-response.

Patients were randomized (2:1:1:2) to receive a placebo intravenously every 4 weeks (*n* = 64), mirikizumab 200 mg (*n* = 31), 600 mg (*n* = 32) and 1000 mg (*n* = 64) intravenously every 4 weeks until week 12. From week 12, patients entered the maintenance phase. Patients who showed improvement (at least a 1-point decrease in SES-CD at week 12 compared to baseline) were re-randomized (1:1) to receive the same dose of intravenous mirikizumab as in the induction phase plus subcutaneous placebo every 4 weeks (IV-C) (*n* = 41) or subcutaneous mirikizumab 300 mg plus intravenous placebo every 4 weeks (IV/SC) (*n* = 46). In contrast, patients who did not experience improvement were not randomized and were instead given mirikizumab 1000 mg intravenously, along with a subcutaneous placebo every 4 weeks (NI/1000 mg) (*n* = 30). The group that received a placebo during the induction phase was also not randomized and received mirikizumab 1000 mg intravenously combined with a subcutaneous placebo every 4 weeks (P/1000 mg) (*n* = 59). Treatments were continued until week 52.

At week 12, 43.8% of patients who received a 1000 mg dose of mirikizumab achieved endoscopic response (a 50% reduction in baseline SES-CD) (*p* = 0.001), while 20.3% achieved endoscopic remission (SES-CD < 4 in ileocolic CD and < 2 in ileal CD) (*p* = 0.009). These findings highlight the efficacy of mirikizumab in treating CD. In contrast, PRO response (reduction ≥ 30% in FS and/or PA and no worsening from baseline), PRO remission (FS ≤ 2. 5 and PA ≤ 1), CDAI response (CDAI < 150 or reduction ≥ 100 points from baseline) and CDAI remission (CDAI < 150) were mainly achieved by the mirikizumab 600 mg group (68.8% (*p* = 0.002), 28.1% (*p* = 0.005), 56.3% (*p* = 0.001) and 40.6% (*p* = 0.001), respectively). Patients receiving the 1000 mg dose of the drug had greater reductions in hs-CRP and CPF levels compared to baseline (mean reductions of 48.6% (*p* < 0.001) and 76.2% (*p* < 0.001), respectively). Additionally, the percentage of patients achieving a normalized CRP (≤3 mg/dL) was also higher in patients treated with this dose (33.3% (*p* < 0.05)).

When these aspects were assessed only in patients who had previously been treated with biologic drugs, the results obtained were lower in those who received 200 mg of the study drug compared with those who had not previously received biologic drugs; however, as the dose was increased, these differences became smaller until they were almost similar at a dose of 1000 mg. However, these findings do not apply to the PRO response, where results are similar at all doses when comparing those who had previously been treated with biologic drugs and those who had not. On the other hand, the CDAI response was higher in those previously treated with biologic drugs for the 200 and 600 mg doses of mirikizumab compared to those not previously treated. Differences in achieving CDAI remission were noted. Superior outcomes were observed in patients treated with doses of 200 and 1000 mg who had not previously received biologic drugs. On the contrary, patients treated with 600 mg achieved better results if they had previously been treated with biologic drugs.

All groups treated with the different doses of the study drug had a greater change in IBDQ score compared to placebo, and the same was true for FS and PA.

Efficacy at week 52 was assessed using the same parameters, and it was observed that the highest percentages of patients achieving endoscopic response, endoscopic remission, PRO response, CDAI response and CDAI remission were in the IV/SC group (58.7%, 32.6%, 71.7%, 69.6% and 56.5%, respectively). In contrast, the highest percentage of patients achieving PRO remission was in the IV-C group (46.3%). The percentage of patients achieving a reduction in hs-CRP from baseline was higher in the IV-C group (59.5%), while for CPF, it was higher in the IV/SC group (81%).

In terms of safety at weeks 0–12, the group with the highest percentage of adverse events was the placebo group (70.3%). The most common adverse events in the study drug groups were headache, worsening CD, arthralgia, nasopharyngitis, weight gain, anemia and nausea. However, the occurrence of these adverse events did not show any correlation with the mirikizumab dosage or the number of patients experiencing them. The placebo group had the highest rate of serious adverse events (10.9%). Among those treated with the study drug, three serious adverse events were reported in the 600 mg dose group (chest pain, worsening of CD and perforation and stricture of the colon) and two in the 1000 mg dose group (abdominal pain and back pain).

Treatment discontinuations due to adverse events were reported, with the highest percentage occurring at the 600 mg dose (9.4%) and none observed at the maximum dose of 1000 mg.

At weeks 12–52, adverse events were the most frequent in the IV/SC group (76.1%), considering only randomized patients.

There were no serious adverse events in the IV-C group, but there were two cases of patients with at least one serious adverse event in the IV/SC group (4.3%). One patient had worsening CD, pyelonephritis and dehydration, and the other had ileal perforation with peritonitis. Both patients discontinued treatment due to worsening CD and ileal perforation. The highest incidence of discontinuation due to side effects was in the IV-C group (2.4%).

No deaths, malignancies, or venous thromboses occurred in either group during this study.

### 3.6. Ustekinumab

This review analyzed the drug ustekinumab through three trials that are derived from the IM-UNITI clinical trial, which was a phase 3, multicenter, randomized, parallel, double-blind, placebo-controlled, multicenter clinical trial. The study will last 44 weeks and is a continuation of the UNITI-1 and UNITI-2 clinical trials. Eligible patients were aged 18–99 years, diagnosed with moderate-to-severe active CD and included those who achieved a clinical response (CDAI ≥ 100) or were in remission at week 8 after receiving intravenous ustekinumab in the two previous clinical trials. These patients were randomized (1:1:1) to receive subcutaneous ustekinumab 90 mg every 8 weeks (*n* = 354), every 12 weeks (*n* = 213) or a subcutaneous placebo (*n* = 151) until week 40. Efficacy and safety were assessed at week 44. Patients who completed these assessments and were deemed by the investigator to benefit from continued treatment were enrolled in a long-term extension study and continued to receive the same dose until week 272 (subcutaneous placebo (*n* = 61), subcutaneous ustekinumab 90 mg every 12 weeks (*n* = 84), subcutaneous ustekinumab 90 mg every 8 weeks (*n* = 82)), every 8 weeks (*n* = 82), every 8 weeks after prior dose adjustment (*n* = 71) (between weeks 8 and 32, patients with loss of response (CDAI ≥ 220 or increase ≥ 100 points from week 0) underwent dose adjustment, but no dose adjustment was performed in the extension study).

Sandborn WJ et al. [51] evaluated efficacy at week 44 in their study using clinical remission (CDAI < 150) as the main criterion. The study found that the drug was effective, with remission rates of 77.4% for patients who took it every 12 weeks and 84.1% for those who took it every 8 weeks.

At week 92, clinical remission was analyzed for efficacy, and the results indicated a decrease in the percentage of patients achieving the target in both the 12-week and 8-week groups (72.6% and 74.4%, respectively). In the pre-titration group, 53.5% of patients achieved clinical remission at week 92.

On the other hand, the percentage of patients who had achieved clinical remission at week 92 was maintained in UNITI-1 and 2:59.4% compared to 80.8% in those treated every 12 weeks, 70.4% compared to 76.4% in those treated every 8 weeks and 46.9% compared to 59% after prior dose titration.

Clinical response (reduction in CDAI ≥ 100 points from week 0 of induction or CDAI < 150) was also assessed and was achieved by 83.3% of those receiving the study drug every 12 weeks, 80.5% of those receiving the study drug every 8 weeks and 67.6% of those who had a previous dose adjustment.

Furthermore, the achievement of clinical remission was evaluated in patients not receiving corticosteroids (CDAI < 150 in patients who had not undergone corticosteroid treatment in the seven days leading up to the visit). Clinical remission was achieved by 67.9% in the 12-week group, 63.4% in the 8 week-group and 42.3% after the previous dosage adjustment.

The CDAI score decreased by 34, 40 and 24 points from baseline in the 12-week, 8-week and dose titration groups, respectively.

A comparison was made between the percentage of patients with normal CRP levels (≥3 mg/dL) at week 92 and week 44, wherein a decrease was observed for both the 12-week and 8-week treatments (from 36.1% to 29.5% and from 30.4% to 26.8%, respectively).

Finally, efficacy was assessed based on the change in IBDQ score at week 92 compared to baseline. The proportion of patients demonstrating an improvement of ≥16 IBDQ points was 73.8% in those treated every 12 weeks, 76.8% in those treated every 8 weeks and 62% after prior dose adjustment. The mean change in each group was 3.5 points in the first group, 9 points in the second group and 6 points in the third group.

The efficacy was analyzed after 252 weeks in a study conducted by Sandborn WJ et al. [52]. In an intention-to-treat analysis of patients randomized to maintenance (IM-UNITI), 28.7% of those treated every 12 weeks and 34.4% of those treated every 8 weeks achieved clinical remission (CDAI < 150).

Including all randomized patients in the long-term extension study, the clinical remission data were modified to 45.2% and 54.9% in the 12-week and 8-week treatment arms, respectively, of which 89.5% and 93.9%, respectively, were not treated with corticosteroids as a result of a tapering schedule for these drugs.

Clinical remission was also analyzed according to whether or not patients had previously been treated with anti-TNF drugs. The results were as follows: in those who had not received anti-TNF drugs, the target was achieved in 28.3% of the group treated every 12 weeks and 44.2% of those treated every 8 weeks, while in patients who had received anti-TNF drugs but did not respond adequately after 5 years of therapy, clinical remission was achieved in 22.8% and 21.4%, respectively.

The study by Li K et al. [53] was based on the IM-UNITI clinical trial and assessed efficacy by histological examination in the first 44 weeks.

Efficacy at week 8 based on the GHAS parameter (mean +/− standard deviation) from baseline showed a change from 10.4 +/− 7 to 7.1 +/− 5.9 (*p* < 0.001) in the ustekinumab group and from 9.2 +/− 6.4 to 7.8 +/− 6.2 (*p* = 0.193) in the placebo group, although this was not statistically significant in the placebo group.

At week 44, patients were assessed for efficacy using the same assay. The results indicate that among randomized patients, those receiving treatment every 8 weeks experienced a change from 7.4 +/− 7.7 to 6.1 +/− 4.7 (*p* > 0.05); those receiving treatment every 12 weeks experienced a change from 5.3 +/− 3.9 to 8.7 +/− 4.1 (*p* > 0.05); and the placebo group experienced a change from 9.2 +/− 3.8 to 10.9 +/− 7.1 (*p* > 0.05).

Histological response (≥50% reduction in GHAS from baseline) was also analyzed in randomized patients. The study drug administered every 8 weeks achieved a histological response in 50% of patients (*p* = 0.0137), while treatment every 12 weeks resulted in a response in 17% of patients (*p* = 0.3529). In contrast, no patients in the placebo group achieved a response.

### 3.7. Risankizumab

The study by Visvanathan S et al. [54] was based on a 12-week, multicenter, randomized, parallel, double-blind, placebo-controlled, double-blind phase 2 clinical trial of those who had been diagnosed with CD for a minimum of three months and exhibited moderate to severe symptoms (CDAI 220–450) upon screening. Patients with ulcers in the mucosa of the ileum and/or colon and a CDEIS ≥ 7 or ≥ 4, if CD was exclusively ileal, were also eligible for enrollment. The study included patients who had received one or more anti-TNFs as well as those who were anti-TNF naive.

Patients were randomized (1:1:1) to receive intravenous risankizumab 200 mg (*n* = 37), 600 mg (*n* = 37) and intravenous placebo (*n* = 32) at weeks 0, 4, 8 and 12.

Efficacy at week 12 was analyzed by risankizumab-induced transcriptome changes (*p* ≤ 0.05) in the colon relative to the ileum compared to baseline. Placebo showed no changes, while the study drug demonstrated a reduction in the expression of 1880 inflammatory genes associated with CD pathogenesis in the colon and 765 genes in the ileum (*p* < 0.005). Some of these genes are *S-100 A8*, *S-100 A9*, *IL8*, *MMP1*, *IFNG*, *LCN2*, *TIMP1*, *TNF*, *STAT3*, *S100 A12* and *MMP3*.

The relationship between endoscopic remission (CDEIS 0–2 points) and the transcriptomic profile was also analyzed and the following changes in the transcriptome from baseline were observed in those who achieved endoscopic remission: 805 downregulated (*p* = 0. 05) and 801 upregulated (*p* = 0.05) genes in those treated with the 600 mg dose, 344 upregulated and 843 downregulated (*p* = 0.05) genes at 200 mg and 152 downregulated (*p* < 0.05) and 33 upregulated (*p* < 0.05) genes in those treated with risankizumab compared to placebo. Some of the downregulated genes include *IL26*, *MMP3*, *S-100 A8* and *S-100 A12*.

Patients who received the study drug and achieved remission or endoscopic response exhibited a pronounced decrease in CD-related gene expression in colon biopsies by week 12 compared to baseline, unlike those who failed to achieve remission or an endoscopic response. In contrast, patients in the placebo group showed increased expression of these genes.

For patients with deep ulcers at baseline, a higher proportion of patients treated with study drug (both doses) achieved an endoscopic response at week 12 than those treated with placebo. Furthermore, these individuals demonstrated a decrease in the expression of genes linked to active CD.

The association between the reduction in fecal biomarkers and the reduction in expression of genes involved in CD at week 12 compared to baseline was also analyzed: 74.4% (*p* = 0.0238) of those treated with the 600 mg dose who had decreased CPF levels had decreased expression of the S-100 A8 gene in the colon and ileum, while 69% of those receiving this dose who had decreased lactoferrin levels had increased expression of this gene. In the placebo group, the decrease was seen in 2.5% and 18.4%, respectively. There were also significant correlations in this group of patients between changes in CPF (*p* = 0.0059) and lactoferrin (*p* = 0.0007) and changes in CDEIS score at week 12 from baseline.

The comparison of tissue and fecal micro-RNA levels at week 12 compared to baseline yielded the following results: 18 microRNAs were differentially expressed in the group receiving a dosage of 600 mg, with 13 exhibiting downregulation and 5 demonstrating upregulation (*p* < 0.05).

### 3.8. PF-00547659

The drug PF-00547659 was analyzed in this systematic review using two studies based on the OPERA clinical trial, which was a phase 2, multicenter, randomized, parallel, double-blind, placebo-controlled, 12-week clinical trial. Patients selected were aged 18–75 years, diagnosed with moderate-to-severe colonic or ileocolic CD, with a CDAI score of 220–450, and ulcers found on colonoscopy in the 8 weeks prior to study screening. Patients included in the study had previously experienced failure or intolerance to immunosuppressive and/or anti-TNF therapy and exhibited a hs-CRP concentration above the upper limit of normal (3 mg/dL). Those who had received >20 mg/day of prednisone (or equivalent oral dose) or >6 mg/day of oral budesonide in the 2 weeks prior to randomization or other biologic drugs, including anti-TNFs, within 6 weeks of randomization were excluded.

A total of 265 patients were enrolled and randomized into four groups (1:1:1:1) to receive placebo (*n* = 64), 22.5 mg (*n* = 68), 75 mg (*n* = 65) and 225 mg (*n* = 68) by subcutaneous injection at weeks 0, 4 and 8 and followed up to week 12. However, one patient in the placebo group and two in the experimental group (22.5 mg) were randomized but not treated.

Efficacy was assessed at weeks 0, 2, 4, 6, 8, 10 and 12; samples were collected for laboratory analysis; neurological assessment was performed; and adverse events and concomitant medication use were recorded.

The efficacy and safety of the treatment were evaluated in a study conducted by Sandborn WJ et al. [55] at weeks 8 and 12 (CI = 90%), respectively. CDAI-70 response (a reduction in CDAI of ≥70 points from baseline), CDAI-100 response (a reduction in CDAI of ≥100 points from baseline) and CDAI remission (CDAI < 150) were used to evaluate efficacy at week 8. The CDAI-70 target was achieved by 52.7% of those treated with 22.5 mg of the drug, 60.1% with the 75 mg dose, 62.7% with the 225 mg dose compared to 47.7% of patients in the placebo group; the CDAI-100 target was achieved by 50.5%, 48.3%, 57% and 41.4%, respectively; and the CDAI remission target was achieved by 29.1% of those treated with 22.5 mg of the drug, 23.8% with the 75 mg dose, 26.9% with the 225 mg dose compared to 16.7% in the placebo group.

At week 12, CDAI-70 response was achieved by 62% of those treated with the 22.5 mg dose, 64.7% of those treated with the 75 mg dose and 57.5% of those treated with the 225 mg dose, compared to 58.6% in the placebo group. CDAI-100 responses were observed in 56%, 47.7%, 53.8% and 44.4%, respectively. Additionally, CDAI remission was achieved in 26.8%, 28.5%, 29.6% and 23%, respectively. However, the significance levels of CDAI and CDAI-100 remission were not statistically significant, unlike the CDAI-70 response.

Changes from baseline in some biomarkers, such as FPC concentration, serum hs-CRP concentration and serum soluble MAdCAM concentration (CI = 90%), were also analyzed in the same week.

A noted reduction in hs-CRP concentration occurred with all drug dosages, particularly with the 22.5 mg dose (36.4%). The serum hs-CRP concentration decreased among patients treated with PF-00547659, with the most significant decrease observed at the 22.5 mg dose (30.9%), whereas it increased by 5.6% in the placebo group.

A decrease in median soluble MAdCAM concentration was observed with all three doses of the study drug, with the greatest decrease at the 225 mg dose (97.8%); however, an increase of 6.7% was observed in the placebo group.

In terms of safety at week 12, adverse events were similar in all groups, with no evidence of an increase in the number of cases with increasing doses of study drug. The highest proportion of adverse events (86.4%) and serious adverse events (16.7%) were reported at the 22.5 mg dose. However, these were considered unlikely to be due to the drug. The 75 mg group had a higher incidence of dose reductions or temporary discontinuation due to adverse events (4.6%), while the 22.5 mg (13.6%) and 75 mg (12.3%) groups had a higher incidence of permanent discontinuation due to adverse events. Out of the 35 patients who had to discontinue treatment, 25 had to do so due to adverse events, with 13 being in the 22.5 mg group and 12 in the 75 mg group. These two groups showed a higher rate of adverse event-related discontinuation, with nine and eight cases, respectively, whereas the 225 mg dose group and the placebo group showed only four such cases each. Most of these dropouts were considered to be due to CD or its complications, with no identified correlation to the medication or increased dosage.

The study by Hassan-Zahraee M et al. [56] assessed efficacy at week 12 compared to baseline and found that serum levels of soluble MAdCAM showed the greatest geometric mean percentage reduction (CI = 90%) from baseline in the 225 mg dose group (97.7%), while an increase of 5.8% was observed in the placebo group.

An increase in hs-CRP levels was observed in the placebo group (from 18.9 mg/dL to 19.9 mg/dL); however, a decrease in these levels was observed in all study drug-treated patients, with the 22.5 mg dose showing the greatest decrease (from 21.1 mg/dL to 11.8 mg/dL) and the 225 mg dose showing the smallest decrease (from 17.2 mg/dL to 15.6 mg/dL).

CPF values, measured in µg/g, showed a decrease in the placebo group and in the 22.5 and 75 mg doses (with the greatest decrease in the 22.5 mg dose: from 1705 to 987 µg/g), while the 225 mg dose showed an increase in CPF values (from 1346 to 1769 µg/g). Finally, transcriptional changes were analyzed in terms of increased expression of the CCR9 gene (encoding proteins that help maintain the pro-inflammatory/anti-inflammatory balance in the gut so that a decrease in its expression leads to a shift in this balance in favor of inflammation). The results showed that all drug doses caused a rise in gene expression, with the increase becoming progressively higher as the dosage increased. There was also an increase observed with the placebo, but it was lower than the increase observed with any of the drug doses.

The results of the trials analyzed are presented in detail in Supplementary Materials.

## 4. Discussion

This review aims to characterize the relationship between different doses of biologic drugs and efficacy and their effectiveness in adult patients diagnosed with moderate-to-severe CD. Patients included had been treated with conventional medicines or even other biologic medication or other biologic agents prior to the trial. These participants either had an inadequate response to treatment or were intolerant to it.

All the trials included in this systematic review showed the efficacy of biologic drugs for moderate CD. Patients receiving upadacitinib, vedolizumab, adalimumab or ustekinumab who were initially on corticosteroids were predominantly able to gradually reduce their intake, successfully addressing corticodependence and cortico-resistance and achieving set targets.

Regarding the safety of the biologic drugs studied, the most common adverse reactions were gastrointestinal (particularly worsening of CD), arthralgia, nausea, headache and nasopharyngitis. Most of these were considered to be mild, and no significant adverse events were observed. Consequently, the biologic drugs were largely well-tolerated, exhibiting a favorable safety profile.

It should be noted that there are specific considerations for each drug during both the induction and maintenance periods.

Treatment with upadacitinib has been shown to be effective in the treatment of moderate–severe CD, as demonstrated in the study by Sandborn WJ et al. [44]. A statistically significant improvement was observed both clinically and endoscopically, with the 6 mg and 24 mg/12 h doses showing the best results at week 16 and the 6 mg and 12 mg/12 h doses showing the best results at week 52.

The clinical improvement involves a reduction in the frequency of liquid stools, relief of abdominal pain, a decrease in the use of drugs to treat diarrhea and a decrease in complications arising from the condition such as luminal stenosis, perforation, arthralgias, abscess or fistula. Furthermore, a statistically significant decrease in serum hs-CRP concentrations indicated a reduction in mucosal inflammation.

Similarly, the work of Mohamed MEF et al. [46] showed good results of upadacitinib in the treatment of this disease, as clinical and endoscopic improvements were observed in patients receiving the drug compared to placebo at week 12, with the 24 mg/12 h dose showing the best results.

In addition, Peyrin-Biroulet et al. [45] conducted a study that confirmed the usefulness of the investigated drug in enhancing the quality of life of patients suffering from this disease. The study revealed a statistically significant improvement in patients’ quality of life as determined by the IBDQ, ED-D5 and WPAI questionnaires. This suggests that patients experience a decrease in severity of discomfort or pain, reduced levels of anxiety or depression and enhanced mobility with greater capability to undertake everyday activities.

The drug was well tolerated and had a good safety profile in terms of adverse events. At week 16, the lowest incidence of serious adverse events and discontinuations were observed with the 6 mg/12 h dosage. However, the highest incidence of adverse events was recorded with the 24 mg/24 h dosage at week 52, whereas the highest number of discontinuations was observed with the 12 mg/12 h dosage.

According to the study by Vermeire S et al. [47], vedolizumab treatment in the maintenance phase improved both clinical remission and clinical response, although the latter did not reach statistical significance compared to the placebo group. Greater clinical remission was also observed in patients previously treated with corticosteroids who tapered off during the study, demonstrating that maintenance treatment with subcutaneous vedolizumab is useful in reducing corticosteroid consumption and side effects in CD patients.

Vedolizumab was generally well tolerated in terms of safety, with decreased frequency rates of mild and serious adverse events compared to placebo, although there was a higher incidence of serious infections.

Adalimumab in the study of D’Haens GR et al. [48] showed that while the HIR dosage achieved higher serum drug concentrations than the SIR dosage during the induction period, this did not translate into a statistically significant endoscopic efficacy, in contrast to the clinical efficacy where the results were statistically significant. Thus, although there was clinical improvement with drug administration, there was no significant change in endoscopic aspects compared to baseline. Similarly, there were no statistically significant differences in clinical or endoscopic parameters during the maintenance period, although the plasma adalimumab concentrations were similar in both the CA and TDM regimens. Nevertheless, a substantial number of patients maintained the favorable response achieved with induction therapy.

Regarding safety, the TDM regimen had the highest incidence of adverse events and discontinuations.

Treatment with guselkumab in patients with moderately active CD in the study by Sandborn WJ et al. [49] was significantly associated with improved clinical and endoscopic outcomes and biomarkers associated with intestinal mucosal inflammation compared with the placebo group, demonstrating the efficacy of the drug in the treatment of CD. The differences observed between the different doses of the study drug were small and not considered clinically significant, and no dose–response relationship was observed. The highest response rate to guselkumab was seen in patients who had previously received conventional treatment, although a high proportion of patients who had previously received biologic drugs responded adequately to the study drug.

In terms of safety, guselkumab had an adequate safety profile with no deaths during the study and generally few serious side effects, with only 1.4% of serious infections occurring.

Mirikizumab in the study by Sands BE et al. [50] showed statistically significant changes that were beneficial at the endoscopic, clinical and biomarker levels, demonstrating that it is an effective drug for the treatment of CD. In induction therapy, the improvement in endoscopy and biomarkers was directly related to the dose increase, with the 1000 mg dose showing the best results. PRO and CDAI results also showed a similar trend, with most patients achieving clinical remission of CD with increasing doses, with the exception of the 1000 mg dose. Maintenance results were similar in both the IV-S and IV/SC groups.

With regard to safety, during the induction period, the placebo group experienced more adverse events and serious adverse events than the mirikizumab group. However, discontinuation was higher among patients receiving the 600 mg study drug dose. Data from both groups were alike during the maintenance phase.

The study conducted by Sandborn WJ et al. [51] demonstrated the potential benefits of ustekinumab treatment in patients with moderate CD, evidenced by a statistically significant improvement in clinical, biomarker and quality of life outcomes at week 92. Notably, this clinical benefit was less pronounced in patients who received dose adjustment, which may be due to the fact that patients had worse quality of life and clinical status prior to dose adjustment than those who did not receive dose adjustment. This suggests a subgroup with more severe disease. Similarly, another study by Sandborn WJ et al. [52] also found clinical improvement in study drug-treated patients compared to placebo at week 252; however, these results were worse than at week 92 because the proportion of patients in clinical remission decreased by 5% each year during the long-term extension study due to treatment discontinuation. At week 252, the patients with the best clinical outcomes were those who had previously been treated with corticosteroids but who had tapered off corticosteroids.

Additionally, Li K et al. [53] demonstrated that the drug was effective in treating CD, with patients receiving the drug exhibiting histological improvement and reducing their GHAS score from baseline. However, in this case, the results were only statistically significant in the eighth week. As a lower baseline GHAS is associated with a higher rate of mucosal healing, patients who take longer to achieve histological improvement may have more severe mucosal involvement or be relatively resistant to ustekinumab.

The Visvanathan S et al. [54] study demonstrated that treatment with risankizumab led to a statistically significant decrease in CD-related gene expression, resulting in improved CD. This improvement was more pronounced in the colon than in the ileum and in individuals with reduced fecal biomarkers (CPF and lactoferrin). The risankizumab-induced association between endoscopic remission and modulation of several genes related to the inflammatory response showed significant changes in the up- or down-expression of these genes, but the authors note that further studies are needed to clarify these changes.

The newer biologic PF-00547659 in the study by Sandborn WJ et al. [55] showed that although overall clinical remission and reduced inflammation by biomarker reduction were not statistically significant, statistically significant results occurred in patients with baseline hs-CRP concentrations > 5 mg/L. It should also be noted that remission and response rates in the placebo group were higher than expected.

The authors suggest a possible methodological failure due to a selection bias, as the proportion of patients treated with anti-TNF was much higher than those not treated with these drugs, or a masking bias, as the colonoscopy assessment performed prior to inclusion in the study was performed without masking, whereas this has been observed to cause increased response and remission in the placebo group in other studies. In addition, in order to avoid performing a biopsy to assess MAdCAM-1 overexpression in CD patients taking the study drug due to the implications and consequences at different levels, a test was developed to measure serum soluble MAdCAM concentrations in the blood as a surrogate marker for the target. This may influence the results or may not be fully representative of the target marker.

The absence of efficiency observed in this study suggests that MAdCAM-1 blockade might only be effective in superficial colonic disease, as there have been studies on the efficacy of this drug in UC with clinically and statistically significant results, inviting further research on this topic [57].

However, in the study by Hassan-Zahraee M et al. [56], the drug proved to be effective as a statistically significant increase in CCR9 gene expression was observed at week 12 compared to baseline. This indicates a reduction in mucosal inflammation, resulting in an improvement in CD. In addition, there was a statistically significant reduction in serum MAdCAM levels at all doses of the study drug, suggesting a reduction in the passage of inflammatory cells from the intravascular space to the intestinal mucosa.

In terms of safety, all three doses of PF-00547659 studied were safe and well tolerated, with most adverse events considered to be related to the underlying disease and not to the study drug.

As with anti-TNF drugs, there is a noteworthy proportion of patients who exhibit inadequate response to the treatment with biologic drugs. This refractoriness, coupled with the high cost of this therapy, leads to the selection of patients who are most likely to respond adequately.

Furthermore, it should be noted that various biologic drugs have different routes of administration. The oral (upadacitinib) and subcutaneous routes (vedolizumab, adalimumab, ustekinumab and PF-00547659) have advantages over the intravenous route (guselkumab, mirikizumab, risankizumab) because they can be administered autonomously by the patient with minimal health education, in the case of the subcutaneous route using autoinjectors; however, the intravenous route requires hospital care, which is associated with higher economic costs, care burden, loss of patient autonomy and therefore a deterioration in quality of life. However, its high price is offset by its efficacy in reducing direct costs of hospital care (both primary care and emergency or inpatient admissions), direct non-medical costs (including special diets and transport to health services) and indirect costs due to improvements in quality of life and work activity [58,59].

The main strength of this systematic review is the analysis of efficacy using different clinical, endoscopic, histological, biomarker, genetic and quality-of-life parameters, which provides a multidimensional view and a global perspective of the patient.

The main limitations found in the studies included in this systematic review are the following. Furthermore, there would be a small sample size used in some of the studies. Some of the biologic drugs were used in combination with others, implying that the observed results cannot be exclusively attributed to the intervention. On the other hand, a value reflecting statistical significance was not available in all trials. In addition, some of the trials that included mucosal biopsies did not include samples from areas not affected by CD, so the effect of treatment on healthy mucosa cannot be assessed. Finally, many of the studies did not collect detailed information about adverse events or reasons for abandoning treatment. Therefore, the conclusions obtained in terms of safety are reduced, which limits their interpretation.

## 5. Conclusions

All eight biologics showed an overall good efficacy profile with statistically significant improvements in clinical, endoscopic, histological, genetic, inflammatory biomarkers and quality of life. Biologic drugs are commonly prescribed as an alternative therapy, but their administration may need to be individualized based on the patient’s condition. Combining them with other treatments can prove beneficial in certain cases.

Treatment with upadacitinib, vedolizumab and ustekinumab allowed the discontinuation of corticosteroids in the majority of patients who were receiving this treatment prior to study entry. This suggests that these drugs may be valuable in maintenance therapy. They may help to reduce the need for corticosteroids and have a favorable safety profile.

Guselkumab, on the other hand, can be considered a fast-acting drug, with a sustained substantial improvement by week 12.

It appears to be an effective treatment for CD, evidenced by the statistically significant differences in treatment observed when administered to patients with higher levels of inflammation compared to the placebo PF-00547659.

Furthermore, most of the patients tolerating this drug report mild side effects, indicating a good safety profile. Common side effects include gastrointestinal symptoms such as exacerbation of CD, headache, arthralgia, nausea and nasopharyngitis.

Therefore, they are postulated to be useful drugs for maintenance therapy due to their corticosteroid-sparing effect and good safety profile.

The results obtained for the different biologic drugs studied highlight the importance of an adequate characterization and multidimensional study of patients with CD (quality of life, clinical, endoscopic, histological, biomarker and transcriptomic profile) to generate hypotheses that can support research on novel treatment approaches with the goal of achieving better disease control and enhancing patients’ quality of life.

Since multiple inflammatory pathways are activated in the inflamed intestine, simply blocking one of them may not be adequate to effectively control inflammation, as is currently carried out with targeted monotherapies. As a result, in the future, it will be necessary to explore alternative approaches. It is necessary to establish treatment strategies, such as sequential or combination therapy, to enhance the efficacy of each medication. These strategies should be objectively evaluated, and their impact on patient outcomes should be comprehensively assessed.

## Figures and Tables

**Table 1:** Inclusion criteria based on PICO algorithm.

Patient (P)	Adult patients (>18 years) with moderate–severe Crohn’s disease
Intervention (I)	Treatment with biologic drugs
Comparison (C)	Placebo, standard care, another dosage regimen, any other drug treatment
Outcome (O)	Daily frequency of loose/very soft stools (SF), daily abdominal pain (AP), Crohn’s Disease Activity Index (CDAI) and Patient Reported Outcomes (PRO); Endoscopic such as the Simple Endoscopic Score for Crohn’s Disease (SES-CD) and the Crohn’s Disease Endoscopic Severity Index (CDEIS); biomarkers such as serum concentration of high-sensitivity C-reactive protein (hs-CRP), C-reactive protein (CRP), lactoferrin, MAd-CAM and fecal calprotectin (FCP); Quality of life through the Inflammatory Bowel Disease Questionnaire (IBDQ), European Quality of Life 5 Dimensions Visual Analog Scale (EQ-D5 VAS), Work Productivity and Activity Impairment (WPAI); Histological, such as the Global Histological Activity Score (GHAS), and genetic, such as changes in the transcriptomic profile. We also included studies that assess safety outcomes as measured by incidence and severity of adverse reactions

## Data Availability

Data is contained within the article and Supplementary Material.

## Supplementary Materials

The following supporting information can be downloaded at https://www.mdpi.com/article/10.3390/ph16111581/s1. Table S1. Efficacy and safety results of upadacitinib. Table S2. Efficacy and safety results of vedolizumab. Table S3. Efficacy and safety results of adalimumab. Table S4. Efficacy and safety results of guselkumab. Table S5. Efficacy and safety results of mirikizumab. Table S6. Efficacy and safety results of ustekinumab. Table S7. Efficacy and safety results of risankizumab. Table S8. Efficacy and safety results of PF-00547659.
