# Supplementary material for: The Efficacy and Safety of Biologic Drugs in the Treatment of Moderate–Severe Crohn’s Disease: A Systematic Review
Source: Pharmaceuticals (Basel). 2023 Nov 8;16(11):1581. doi: 10.3390/ph16111581 (PMC10674451; doi:10.3390/ph16111581)
Supplement: Supplementary file 1 [file pharmaceuticals-16-01581-s001.zip › pharmaceuticals-2665077-supplementary.pdf]

Supplementary Materials. Summary of efficacy and safety results for each biological drug analyzed

Table S1. Efficacy and safety results of **upadacitinib**

Reference (Trial identification)	Design	Phase	Intervention group	Control group	Treatment period	Effectiveness	Security
Sandborn WJ et al. 2020 [44]<br>CELEST<br>(NCT02365649)	- Multicentric<br>- Randomized<br>- Placebo controlled<br>- Double-blind<br>- Parallel	2	**Age:** 18-75 years<br><br>Weeks 0-16:<br><br>-Upadacitinib oral 3mg/12h (n=39)<br><br>-Upadacitinib oral 6mg/12h (n=37)<br><br>-Upadacitinib oral 12mg/12h (n=36)<br><br>-Upadacitinib oral 24mg/12h (n=36)<br><br>-Upadacitinib oral 24mg/24h (n=35)<br><br>Weeks 16-52:<br><br>-Upadacitinib oral 6mg/12h (n=23)<br><br>-Upadacitinib oral 12mg/12h (n=59)<br><br>-Upadacitinib oral 24mg/24h (n=37)	**Age:** 18-75 years<br><br>Weeks 0-16:<br><br>-Placebo (n=37)<br><br>Weeks 16-52:<br><br>-Upadacitinib oral 3mg/12h (n=61)	52 weeks	**Clinical remission 1.5/1.0 (w16) (P//3//6//12//24//24.):**<br>11%//13%//27%//11%//22%//14%	**Adverse events (w16) (P//3//6//12//24//24.):**<br>73%//87.2%//78.4%//80.6%// 83.8%//82.9%
						**Clinical remission 2.8/1.0 (w16) (P//6//24):**<br>12%//30%//37%	
						**Clinical remission 1.5/1.0 in those not treated with corticosteroids (w16) (P//24):**<br>0%//33%	**Serious adverse events (w16) (P//3//6//12//24//24.):**<br>5.4%//12.8%//5.4%//27.8%//8. 3%//20%
						**CDAI<150 (w16) (P//U):**<br>16%//20-39%	
						**CDAI<150 in those treated with corticosteroids (w16) (P//12//24):**<br>0%//41%//33%	**Adverse events leading to discontinuation (w16) (P//3//6//12//24//24.):**<br>13.5%//10.3%//2.7%//25%//8.3 %//11.4%
						**hs-CRP (mg/l) Median decrease (w16/RB) (P//3//6//12//24//24.):**<br>0//0//4.6//0.3//3.2//0.2	
						**Clinical remission 2.8/1.0 (w52) (R/DR) (3//6//12//24.):**<br>41/29%//63/43%//73/52%//40/39%	**Adverse events (w52) (3//6//12//24.):**<br>75%//60.9%//72.9%//63.9%
						**Endoscopic response 50% (w52) (R/DR) (3//6//12//24.):**<br>50/34%//50/36%//69/45%//30/37%	**Serious adverse events (w52) (3//6//12//24.):**<br>25%//8.7%//8.5%//11.1%
						**CDAI<150 (w52) (R/DR) (3//6//12//24.):**<br>55/44%//50/50%//69/55%//40/37%	**Adverse events leading to discontinuation (w52) (3//6//12//24.):**<br>10%//0%//8.5%//8.3%
						**CDAI<150 in those not treated with corticosteroids (w52) (R/DR) (3//6//12//24.):**<br>50/47%//100/67%//63/57%//0/13%	

w16: week 16; P: placebo; 3: upadacitinib 3mg/12h; 6: upadacitinib 6mg/12h; 12: upadacitinib 12mg/12h; 24: upadacitinib 24mg/12h; 24.: upadacitinib 24mg/24h; CDAI: Crohn's disease Activity Index; hs-CRP: high-sensitivity C-reactive protein; RB: respect to the baseline; w52: week 52; R: responders; DR: doubly responders

Table S1. Efficacy and safety results of **upadacitinib** (continuation)

Reference (Trial identification)	Design	Phase	Intervention group	Control group	Treatment period	Effectiveness	
Peyrin-Biroulet L et al. 2021 [45] CELEST (NCT02365649)	- Multicentric<br>- Randomized<br>- Placebo controlled<br>- Double-blind<br>- Parallel	2b	**Age:** 18-75 years<br><br>Weeks 0-16:<br><br>-Upadacitinib oral 3mg/12h (n=39)<br><br>-Upadacitinib oral 6mg/12h (n=37)<br><br>-Upadacitinib oral 12mg/12h (n=36)<br><br>-Upadacitinib oral 24mg/12h (n=36)<br><br>-Upadacitinib oral 24mg/24h (n=35)<br><br>Weeks 16-52:<br><br>-Upadacitinib oral 6mg/12h (n=14)<br><br>-Upadacitinib oral 12mg/12h (n=29)<br><br>-Upadacitinib oral 24mg/24h (n=19)	**Age:** 18-75 years<br><br>Weeks 0-16:<br><br>-Placebo (n=37)<br><br>Weeks 16-52:<br><br>-Upadacitinib oral 3mg/12h (n=32)	52 Weeks	**IBDQ score changes:**<br>Week 8 (P//3//6//12//24//24.):<br>+17//+19//+35//+25//+40//+23<br>Week 16 (P//3//6//12//24//24.):<br>+13//+21//+39//+27//+41//+22<br>Week 52 (3//6//12//24.):<br>+43//+47//+71//+27	**Presenteeism (WPAI):**<br>Week 8 (P//3//6//12//24//24.):<br>-13%//-31%//-16%//-10%//-6%//-8%<br>Week 16 (P//3//6//12//24//24.) (%):<br>-4%//-18%//-23%//-13%//-17%//-3%<br>Week 52 (3//6//12//24.) (%):<br>-32%//-24%//-37%//-20%
						**IBDQ remission:**<br>Week 8 (P//3//6//12//24//24.):<br>16%//23%//30%//28%//36%//23%<br>Week 16 (P//3//6//12//24//24.):<br>11%//23%//32%//33%//39%//26%<br>Week 52 (3//6//12//24.):<br>44%//50%//41%//32%	**Absenteeism (WPAI):**<br>Week 8 (P//3//6//12//24//24.):<br>-2%//+5%//-5%//-14%//+1%//-5%<br>Week 16 (P//3//6//12//24//24.):<br>+4%//+1%//-6%//-15%//+2%//-1%<br>Week 52 (3//6//12//24.):<br>-7%//-23%//-13%//-5%
						**Response IBDQ:**<br>Week 8 (P//3//6//12//24//24.):<br>38%//41%//62%//58%//53%//57%<br>Week 16 (P//3//6//12//24//24.):<br>24%//46%//57%//50%//56%//49%<br>Week 52 (3//6//12//24.):<br>44%//79%//69%//32%	**Overall work impairment (Presenteeism y absenteeism) (WPAI):**<br>Week 8 (P//3//6//12//24//24.):<br>-11%//-21%//-17%//-17%//-6%//-9%<br>Week 16 (P//3//6//12//24//24.):<br>-2%//-17%//-21%//-20%//-16%//-4%<br>Week 52 (3//6//12//24.):<br>-23%//-37%//-38%//-21%
						**EQ-5D VAS score:**<br>Week 8 (P//3//6//12//24//24.):<br>+8//+8//+15//+6//+14//+8<br>Week 16 (P//3//6//12//24//24.):<br>+7//+9//+17//+10//+15//+9<br>Week 52 (3//6//12//24.):<br>+18//+22//+36//+8	**Activity impairment (disability) (WPAI):**<br>Week 8 (P//3//6//12//24//24.):<br>-6%//-14%//-18%//-13%//-14%//-8%<br>Week 16 (P//3//6//12//24//24.):<br>-4%//-11%//-19%//-14%//-21%//-10%<br>Week 52 (3//6//12//24.):<br>-29%//-26%//-42%//-15%

IBDQ: Inflammatory Bowel Disease Questionnaire; P: placebo; 3: upadacitinib 3mg/12h; 6: upadacitinib 6mg/12h; 12: upadacitinib 12mg/12h; 24: upadacitinib 24mg/12h; 24: upadacitinib 24mg/24h; EQ-5D VAS: European Quality of Life-5 Dimensions visual analog scale; WPAI: Work Productivity and Activity Impairment

Table S1. Efficacy and safety results of **upadacitinib** (continuation)

Reference (Trial identification)	Design	Phase	Intervention group	Control group	Treatment period	Effectiveness	Security (Adverse events)
Mohamed MEF et al. 2020 [46]<br>CELEST<br>(NCT02365649)	- Multicentric<br>- Randomized<br>- Placebo controlled<br>- Double-blind<br>- Parallel	2	**Age:** 18-75 years<br><br><u>Weeks 0-16:</u><br><br>-Upadacitinib oral 3mg/12h (n=39)<br><br>-Upadacitinib oral 6mg/12h (n=37)<br><br>-Upadacitinib oral 12mg/12h (n=36)<br><br>-Upadacitinib oral 24mg/12h (n=36)<br><br>-Upadacitinib oral 24mg/24h (n=35)	**Age:** 18-75 years<br><br><u>Weeks 0-16:</u><br><br>-Placebo (n=37)	16 Weeks	**Clinical response (w12) (P//3//6//12//24//24.):**<br>35%//49%//54%//57%//61%//55%<br><br>**Clinical remission 2.8/1.0 (w12) (P//3//6//12//24//24.):**<br>12%//19%//23%//28%//33%//22%<br><br>**CDAI<150 (w12) (P//3//6//12//24//24.):**<br>21%//25%//27%//32%//36%//26%<br><br>**Endoscopic response 25% (w12) (P//3//6//12//24//24.):**<br>15%//26%//34%//45%//57%//45%<br><br>**Endoscopic response 50% (w12) (P//3//6//12//24//24.):**<br>3%//14%//20%//28%//36%//28%<br><br>**Endoscopic remission (w12) (P//3//6//12//24//24.):**<br>0%//6%//10%//15%//20%//15%	**Herpes Zoster (uw16):**<br>-Cave (34,100): 2.2%<br><br>**Pneumonia (uw16):**<br>-Placebo: 2.7%<br>-Cave (20,34): 2.2%<br><br>**Serious infections (uw16)**:<br>-Cave (4,12): 2.3%<br>-Cave (12,20): 6.7%<br>-Cave (20,34): 4.4%<br>-Cave (34,100): 4.4%<br><br>**Lymphopenia ≥ grade 1 (w16):**<br>-Placebo: 21.6%<br>-Cave (4,12): 22.7%<br>-Cave (12,20): 20%<br>-Cave (20,34): 22.2%<br>-Cave (34,100): 28.9%<br><br>**Lymphopenia ≥ grade 2 (w16):**<br>-Placebo: 5.4%<br>-Cave (4,12): 6.8%<br>-Cave (12,20): 8.9%<br>-Cave (20,34): 4.4%<br>-Cave (34,100): 11.1%<br><br>**Lymphopenia ≥ grade 3 (w16):**<br>-Cave (34,100): 4.4%<br><br>**Decreases in hemoglobin ≥ 2g/dl (w16):**<br>-Cave (12,20): 4.4%<br>-Cave (20,34): 4.4%<br>-Cave (34,100): 2.2%

w12: week 12; P: placebo; 3: upadacitinib 3mg/12h; 6: upadacitinib 6mg/12h; 12: upadacitinib 12mg/12h; 24: upadacitinib 24mg/12h; 24.: upadacitinib 24mg/24h; CDAI: Crohn's disease Activity Index; uw16: until week 16; C_{ave}: average plasma concentration (ng/ml); w16: week 16

**Table S2.** Efficacy and safety results of **vedolizumab**							
Reference (Trial identification)	Design	Phase	Intervention group	Control group	Treatment period	Effectiveness	Security
Vermeire S et al. 2022 [47] VISIBLE2 (NCT02611817)	- Multicentric<br>- Randomized<br>- Placebo controlled<br>- Double-blind<br>- Parallel	3	**Age:** 18-80 years<br><br>- Vedolizumab 108mg SC every 2 weeks (n=275)	**Age:** 18-80 years<br><br>- Placebo SC every 2 weeks (n=135)	52 weeks	**Clinical remission (w6):**<br>50.6% of patients treated<br>**Clinical response (w6):**<br>84.4% of patients treated<br>**Clinical remission (w52):**<br>-Vedolizumab: 48%<br>-Placebo: 34.3%<br>**Clinical response (w52):**<br>-Vedolizumab: 52%<br>-Placebo: 44.8%<br>**Clinical remission (w52):**<br>-Vedolizumab: 45.3%<br>-Placebo: 18.2%<br>**Clinical remission in patients not treated with anti-TNF (w52):**<br>-Vedolizumab: 48.6%<br>-Placebo: 42.9%<br>**Clinical remission in patients with failure to treatment with anti-TNF (w52):**<br>-Vedolizumab: 46.4%<br>-Placebo: 28.8%<br>**Clinical remission in patients not treated with anti-TNF (w52):**<br>-Vedolizumab: 41%<br>-Placebo: 18.2%<br>**Clinical remission in patients with failure to treatment with anti-TNF (w52):**<br>-Vedolizumab: 46.2%<br>-Placebo: 15%<br>**FCP ≤ 250µg/g (s52):**<br>-Vedolizumab: 60.5%<br>-Placebo: 31.7%<br>**CRP < 5mg/l (s52):**<br>-Vedolizumab: 23.2%<br>-Placebo: 17.5%	**Adverse events:**<br>-Vedolizumab: 73.5%<br>-Placebo: 76.1%<br>**Adverse events possibly related to treatment:**<br>-Vedolizumab: 19.3%<br>-Placebo: 14.9%<br>**Serious adverse events:**<br>-Vedolizumab: 8.4%<br>-Placebo: 10.4%<br>**Discontinuation of treatment due to adverse events:**<br>-Vedolizumab: 4%<br>-Placebo: 8.2%<br>**Incidence of serious infections:**<br>-Vedolizumab: 86 cases<br>-Placebo: 46 cases<br>**Incidence of deaths:**<br>-Vedolizumab: 0 cases<br>-Placebo: 0 cases

SC: subcutaneous; w6: week 6; w52: week 52; FCP: faecal calprotectin; CRP: C-reactive protein

Table S3. Efficacy and safety results of **adalimumab**

Reference (Trial identification)	Design	Phase	Intervention group	Control group	Treatment period	Effectiveness	Security
D´Haens GR et al. 2022 [48]<br>SERENE (NCT02065570)	- Multicentric<br>- Randomized<br>- Placebo controlled<br>- Double-blind<br>- Parallel	3	**Age:** 18-75 years<br><br><u>Weeks 0-12:</u><br><br>-HIR (n=308): Adalimumab 160mg SC in weeks 0,1,2,3 and 40 mg from week 4 to week 12<br><br><u>Weeks 12-56:</u><br><br>-CA (n=92): increase Adalimumab 40mg SC every week if CDAI score was ≥220 or hs-CRP levels ≥ 10 mg/l	**Age:** 18-75 years<br><br><u>Weeks 0-12:</u><br><br>-SIR (n=206): Adalimumab 160 mg SC in week 0, placebo in week 1, 80 mg in week 2, placebo in week 3 and 40 mg from week 4 to week 12<br><br><u>Weeks 12-56:</u><br>-TDM (n=92):<br>if adalimumab concentration < 5µg/ml: 40mg of adalimumab SC every week;<br>if adalimumab concentration > 10µg/ml: 40mg of adalimumab SC every other week;<br>if adalimumab concentration 5-10µg/ml: 40mg adalimumab SC every week if CDAI score ≥ 220 or hs-CRP levels ≥ 10mg/l	56 Weeks	**Clinical remission (w4) (HIR//SIR):** 43.5%//43.7%<br>**Endoscopic response (w12) (HIR//SIR):** 42.9%//39.3%<br>**Clinical remission (w12) (HIR//SIR):** 62.3%//51.5%<br>**Clinical response (w12) (HIR//SIR):** 83.4%//74.8%<br>**Clinical remission (w56) (CA//TDM):** 70.7%//66.3%<br>**Endoscopic response (w56) (CA//TDM):** 44.6%//43.5%<br>**Endoscopic remission (w56) (CA//TDM):** 31.5%//29.3%<br>**Clinical remission without corticosteroids (w56) (CA//TDM):** 76.9%//73.2%<br>**Deep remission (w56) (CA//TDM):** 29.3%//26.1%	**Adverse events (HIR//SIR//CA//TDM):** 60.1%//64.6%//70.6%//69.7%<br>**Treatment-emergent adverse event possibly related to study drug (HIR//SIR//CA//TDM):** 24.4%//26.2%//26.6%//30.3%<br>**Serious adverse events (HIR//SIR//CA//TDM):** 4.5%//4.9%//4.6%//6.4%<br>**Severe treatment-emergent adverse event (HIR//SIR//CA//TDM):** 5.5%//6.3%//6.4%//5.5%<br>**Mild infections (HIR//SIR//CA//TDM):** 22.4%//23.8%//33.9%//34.9%<br>**Adverse events leading to discontinuation of study drug (HIR//SIR//CA//TDM):** 4.2%//3.9%//7.3%//8.3%<br>**Deaths (HIR//SIR//CA//TDM):** 0%//0%//0%//0%

HIR: higher induction regimen; CA: clinically adjusted; hs-CRP: high-sensitivity C-reactive protein; TDM: therapeutic drug monitoring; CDAI: Crohn’s Disease Activity Index; SIR: standard induction regimen; w4: week 4; w12: week 12; w56: week 56

**Table S4.** Efficacy and safety results of **guselkumab**							
Reference (Trial identification)	Design	Phase	Intervention group	Control group	Treatment period	Effectiveness	Security
Sandborn WJ et al. 2022 [49]<br>GALAXI-1<br>(NCT03466411)	- Multicentric<br>- Randomized<br>- Placebo controlled<br>- Double-blind<br>- Parallel	2	**Age:** >18 years<br><br>-Guselkumab 200mg IV Weeks 0, 4 and 8 (n=61)<br><br>-Guselkumab 600mg IV Weeks 0, 4 and 8 (n=63)<br><br>-Guselkumab 1200mg IV Weeks 0, 4 and 8 (n=61)	**Age:** >18 years<br><br>-Placebo Weeks 0, 4 and 8 (n=61)	12 Weeks	**Changes in baseline CDAI score compared to baseline (w12):**<br>-Placebo: -36.2<br>-Guselkumab 200mg: -160.4<br>-Guselkumab 600mg: -138.9<br>-Guselkumab 1200mg: -144.9	**Adverse events (w12):**<br>-Placebo: 60%<br>-Guselkumab: 45.7%
						**Clinical remission (w12):**<br>-Guselkumab: 53%, 47.5% (PTB), 59.5% (CPT)<br>-Placebo: 16.4%, 10% (TPB) y 22.6% (CPT)	**Serious adverse events (w12):**<br>-Placebo: 5.7%<br>-Guselkumab: 3.7%
						**Clinical response (w12):**<br>-Guselkumab: 65.9%, 62.4% (PTB) y 70.2% (CPT)<br>-Placebo: 24.6%, 20% (PTB) y 29% (CPT)	**Infections (w12):**<br>-Placebo: 21.4%<br>-Guselkumab: 15.1%
						**Remission PRO (w12):**<br>-Guselkumab: 42.7%, 40.6% (PTB) y 45.2% (CPT)<br>-Placebo: 16.4%, 13.3% (PTB) y 19.4% (CPT)	
						**Endoscopic remission (w12):**<br>-Guselkumab: 35.7%, 30.7% (PTB) y 41.7% (CPT)<br>-Placebo: 11.5%, 13.3% (PTB) y 9.7% (CPT)	**Serious infections (w12):**<br>-Placebo: 0%<br>-Guselkumab: 1.4%
						**Clinical biomarker response (w12):**<br>-Guselkumab: 47%<br>-Placebo: 6.6%	**Incidence of deaths:**<br>-Placebo: 0 cases<br>-Guselkumab: 0 cases

IV: intravenous; CDAI: Crohn's Disease Activity Index; w12: week 12; PTB: previous treatment with biologicals; CPT: conventional pretreatment; PRO: Patient Reported Outcome

<table>
<tr><th colspan="8">Table S5. Efficacy and safety results of mirikizumab</th></tr>
<tr><th>Reference (Trial identification)</th><th>Design</th><th>Phase</th><th>Intervention group</th><th>Control group</th><th>Treatment period</th><th>Effectiveness</th><th>Security</th></tr>
<tr><td rowspan="2">Sands BE et al. 2022 [50]
SERENITY

(NCT02891226)</td><td rowspan="2">- Multicentric
- Randomized
- Placebo controlled
- Double-blind
- Parallel</td><td rowspan="2">2</td><td rowspan="2">Age: 18-75 years

Weeks 0-12:

- Mirikizumab 200mg IV every 4 weeks (n=31)

- Mirikizumab 600mg IV every 4 weeks (n=32)

- Mirikizumab 1000mg IV every 4 weeks (n=64)

Weeks 12-52:

- IV-C: same dose of mirikizumab IV than in induction phase + SC placebo every 4 weeks (n=41)

- IV/SC: SC mirikizumab 300mg + placebo IV every 4 weeks (n=46)</td><td rowspan="2">Age: 18-75 years

Weeks 0-12:

- Placebo IV every 4 weeks (n=64)

Weeks 12-52:

- Placebo/1000mg: mirikizumab IV 1000mg + placebo SC every 4 weeks (n=59)</td><td rowspan="2">52 weeks</td><td>Week 12 (P//200//600//1000):
Endoscopic response:
10.9%//25.8%//37.5%//43.8%
Endoscopic remission:
1.6%//6.5%//15.6%//20.3%
PRO response:
35.9%//61.3%//68.8%//60.9%
PRO remission:
6.3%//12.9%//28.1%//21.9%
CDAI response:
23.4%//48.4%//56.3%//42.2%
CDAI remission:
9.4%//16.1%//40.6%//26.6%
Median decrease hs-CRP RB:
43.8%*//29.9%//39.8%//48.6%
Median decrease FCP RB:
0%//60.7%//62.1%//76.2%
CRP ≤ 3mg/l:
9.1%//4.3%//26.1%//33.3%</td><td>Week 12 (P//200//600//1000):
Adverse events:
70.3%//58.1%//65.6%//65.6%
Serious adverse events:
10.9%//0%//9.4%//3.1%
Discontinuation of treatment due to adverse events:
6.3%//3.2%//9.4%//0%
Incidence of deaths:
0%//0%//0%//0%</td></tr>
<tr><td>Week 52 (IV-C//IV/SC):
Endoscopic response:
58.5%//58.7%
Endoscopic remission:
19.5%//32.6%
PRO response:
68.3%//71.7%

PRO remission:
46.3% //45.7%
CDAI response:
53.7%//69.6%
CDAI remission:
39%//56.5%
Median decrease hs-CRP RB:
59.5%//52.4%
Median decrease FCP RB:
78.2%//81%</td><td>Week 52
(IV-C //IV/SC):
Adverse events:
75.6%//76.1%
Serious adverse events:
0%//4.3%
Discontinuation of treatment due to adverse events:
2.4%//2.2%
Incidence of deaths:
0%//0%</td></tr>
</table>

IV-C: combined IV groups; IV: intravenous; SC: subcutaneous; P: placebo; 200: mirikizumab 200mg; 600: mirikizumab 600 mg; 1000: mirikizumab 1000mg; PRO: Patient-Reported Outcome; CDAI: Crohn's Disease Activity Index; hs-CRP: high-sensitivity C-reactive protein; RB: respect to the baseline; FCP: fecal calprotectin; *: median increase; CRP: C-reactive protein

**Table S6.** Efficacy and safety results of **ustekinumab**						
Reference (Trial identification)	Design	Phase	Intervention group	Control group	Treatment period	Effectiveness
Sandborn WJ et al. 2018 [51]<br>IM-UNITI (LTE)<br>(NCT01369355)	- Multicentric<br>- Randomized<br>- Placebo controlled<br>- Double-blind<br>- Parallel	3	**Age:** 18-99 years<br><br>Weeks 0-44:<br><br>-Ustekinumab 90mg SC e12w (n=213)<br><br>-Ustekinumab 90mg SC e8w (n=354)<br><br>Weeks 45-272:<br><br>-Ustekinumab 90mg SC e12w (n=84)<br><br>-Uustekinumab 90mg SC e8w (n=82)<br><br>-Ustekinumab 90mg SC e8w (after dose adjustment) (n=71)	**Age:** 18-99 years Weeks 0-44:<br><br>-Placebo SC (n=151)<br><br>Weeks 45-272:<br><br>-Placebo SC (n=61)	272 Weeks	**Clinical remission (w44) (ustekinumab 90mg e12w//e8w):**<br>77.4%//84.1%<br><br>**Clinical remission (w92) (ustekinumab 90mg e12w//e8w//dose adjustment):**<br>72.6%//74.4%//53.5%<br><br>**Patients who, having achieved clinical remission in IM-UNITI 1 and 2, maintain it at week 92 (ustekinumab 90mg e12w//e8w//dose adjustment): ***<br>80.8%//76.4%//59%<br><br>**Clinical response w92) (ustekinumab 90mg e12w//e8w//dose adjustment):**<br>83.3%//80.5%//67.6%<br><br>**Clinical remission in those not treated with corticosteroids (w92) (ustekinumab 90mg e12w//e8w//dose adjustment):**<br>67.9%//63.4%//42.3%<br><br>**Decrease in CDAI score compared to baseline (w92) (ustekinumab 90mg e12w//e8w//dose adjustment):**<br>34 points //40 points //24 points<br><br>**Decrease in the percentage of patients with hs-CRP ≥ 3 mg/l week 92 compared to week 44 (ustekinumab 90mg e12w//e8w):**<br>From 36.1% to 29.5%// From 30.4% to 26.8%<br><br>**IBDQ improvement ≥ 16 points (w92) compared to score at the beginning of the study (ustekinumab 90mg e12w//e8w//dose adjustment):** 73.8%//76.8%//62%

LTE: long term extension study; SC: subcutaneous; w44: week 44; e12w: every 12 weeks; e8w: every 8 weeks; w92: week 92; *: Patients who achieved clinical remission in IM-UNITI 1 and 2 were 59.4% of those treated every 12 weeks, 70.4% of those treated every 8 weeks and 46.9% of those who received an adjustment of dose; CDAI: Crohn's Disease Activity Index; hs-CRP: high-sensitivity C-reactive protein; IBDQ: Inflammatory Bowel Disease Questionnaire

**Table S6.** Efficacy and safety results of **ustekinumab** (continuation)						
Reference (Trial identification)	Design	Phase	Intervention group	Control group	Treatment period	Effectiveness
Sandborn WJ et al. 2022 [52]<br>IM-UNITI (LTE)<br>(NCT01369355)	- Multicentric<br>- Randomized<br>- Placebo controlled<br>- Double-blind<br>- Parallel	3	**Age:** 18-99 years<br><br>Weeks 0-44:<br><br>-Ustekinumab 90mg SC e12w (n=213)<br><br>-Ustekinumab 90mg SC e8w (n=354)<br><br>Weeks 45-272:<br><br>-Ustekinumab 90mg SC e12w (n=84)<br><br>-Ustekinumab 90mg SC e8w (n=82)<br><br>-Ustekinumab 90mg SC e8w (after dose adjustment) (n=71)	**Age:** 18-99 years<br><br>Weeks 0-44:<br><br>- Placebo SC (n=151)<br><br>Weeks 45-272:<br><br>- Placebo SC (n=61)	272 Weeks	**Clinical remission (w252) (intention-to-treat analysis) (ustekinumab 90mg e12w//e8w):**<br>28.7%//34.4%
						**Clinical remission in randomized patients in LTE (w252) (ustekinumab 90mg e12w//e8w):**<br>45.2% (of which 89.5% were not receiving corticosteroids)//54.9% (of which 93.9% were not receiving corticosteroids)
						**Clinical remission in patients not previously treated with anti-TNF (w252) (ustekinumab 90mg e12w//e8w):**<br>28.3%//44.2%
						**Clinical remission in patients previously treated with anti-TNF (w252) (ustekinumab 90mg e12w//e8w):**<br>22.8%//21.4%

LTE: long term extension study; SC: subcutaneous; w252: week 252; e12w: every 12 weeks; e8w: every 8 weeks; anti-TNF: anti- Tumor Necrosis Factor

**Table S6.** Efficacy and safety results of **ustekinumab** (continuation)						
Reference (Trial identification)	Design	Phase	Intervention group	Control group	Treatment period	Effectiveness
Li K et al. 2019 [53] IM-UNITI (NCT01369355)	- Multicentric<br>- Randomized<br>- Placebo controlled<br>- Double-blind<br>- Parallel	3	**Age:** 18-99 years<br><br>Weeks 0-44:<br><br>-Ustekinumab 90mg SC e12w (n=213)<br><br>-Ustekinumab 90mg SC e8w (n=354)	**Age:** 18-99 years<br><br>Weeks 0-44:<br><br>-Placebo SC (n=151)	44 Weeks	**Changes GHAS (w8) (mean +/- standard deviation):**<br><br>-Ustekinumab: from 10.4 +/- 7 to 7.1 +/- 5.9<br>-Placebo: from 9.2 +/- 6.4 to 7.8 +/- 6.2
						**Changes GHAS (w44) (mean +/- standard deviation):**<br><br>-Ustekinumab 90mg e12w: from 5.3 +/-3.9 to 8.7 +/- 4.1<br>-Ustekinumab 90mg e8w: from 7.4 +/-7.7 to 6.1 +/- 4.7<br>-Placebo: from 9.2 +/-3.8 to 10.9 +/- 7.1
						**Histologic response (w44):**<br><br>-Ustekinumab 90mg e12w: 50%<br>-Ustekinumab 90mg e8w: 17%<br>-Placebo: 0%

SC: subcutaneous; e12w: every 12 weeks; e8w: every 8 weeks GHAS: Global Histology Activity Score; w8: week 8; w44: week 44

Table S7. Efficacy and safety results of risankizumab						
Reference (Trial identification)	Design	Phase	Intervention group	Control group	Treatment period	Effectiveness
Visvanathan S et al. 2018 [54] (NCT02031276)	- Multicentric<br>- Randomized<br>- Placebo controlled<br>- Double-blind<br>- Parallel	2	**Age:** 18-75 years<br><br>-Risankizumab 200mg IV Weeks 0, 4, 8 and 12 (n=37)<br><br>-Risankizumab 600mg IV Weeks 0, 4, 8 and 12 (n=37)	**Age:** 18-75 years<br><br>-Placebo IV Weeks 0, 4, 8 and 12 (n=32)	12 Weeks	**Changes in the transcriptome induced by risankizumab (w12/RB with risankizumab):**<br>-Colon: decrease in the expression of 1880 genes<br>-Ileum: decrease in the expression of 765 genes<br>-No significant changes with placebo
						**Association between endoscopic remission and transcriptomic profile in patients treated with risankizumab vs placebo (w12/RB with risankizumab):**<br>-Risankizumab 600 mg: 805 DR genes and 801 UR genes<br>-Risankizumab 200 mg: 344 UR genes<br>-Risankizumab 200 and 600 mg compared to placebo: 152 DR genes and 33 UR genes
						**Association between transcriptomic profile and changes in fecal biomarkers (w12/RB):**<br>-Risankizumab 600 mg: 74.4% FCP * and 69% of LT**<br>-Placebo: 2.5% FCP * and 18.4% of LT*
						**Changes in miRNAs tissue and faecal (w12/RB with risankizumab):**<br>-Risankizumab 600 mg: 13 DR miRNAs and 5 UR miRNAs<br>-Placebo: not observed DR miRNAs

IV: intravenous; w12/RB: week 12 respect to the baseline; DR: Downregulation; UR: Upregulation; FCP: faecal calprotectin; LT: lactoferrin; *: decrease in the expression of the *S-100A8* gene and the biomarker; **: increase in the expression of the *S-100A8* gene and decrease in the biomarker; miRNA: micro RNA

Table S8. Efficacy and safety results of **PF-00547659**

Reference (Trial identification)	Design	Phase	Intervention group	Control group	Treatment period	Effectiveness	Security
Sandborn WJ et al. 2018 [55]<br>OPERA<br>(NCT01276509)	- Multicentric<br>- Randomized<br>- Placebo controlled<br>- Double-blind<br>- Parallel	2	**Age:** 18-75 years<br><br>-PF-00547659 22.5 mg SC Weeks 0, 4 and 8 (n=68)<br><br>-PF-00547659 75 mg SC Weeks 0, 4 and 8 (n=65)<br><br>-PF-00547659 225 mg SC Weeks 0, 4 and 8 (n=68)	**Age:** 18-75 years<br><br>-Placebo SC Weeks 0, 4 and 8 (n=64)	12 Weeks	**Response CDAI-70 (w8) (P//22.5//75//225):** 47.7%//52.7%//60.1%//62.7%<br>**Response CDAI-100 (w8) P//22.5//75//225):** 41.4%//50.5%//48.3%//57%<br>**Remission CDAI (w8) (P//22.5//75//225):** 16.7%//29.1%//23.8%//26.9%<br>**Response CDAI-70 (w12) (P//22.5//75//225):** 58.6%//62%//64.7%//57.5%<br>**Response CDAI-100 (w12) (P//22.5//75//225):** 44.4%//56%//47.7%//53.8%<br>**Remission CDAI (w12) (P//22.5//75//225):** 23%//26.8%//28.5%//29.6%<br>**GMR of FCP (w12) (P//22.5//75//225):** 6.5%//36.4%//29.4%//10.5%<br>**GMR of hs-CRP (w12) (P//22.5//75//225):** 5.6%*//30.9%//21.8%//20%<br>**MS of soluble MAdCAM concentrations (w12) (P//22.5//75//225):** 6.7%//88.7%//96.5%//97.8%	**Number of adverse events (w12) (P//22.5//75//225):** 141//175//192//190<br>**Adverse events (w12) (P//22.5//75//225):** 85.7%//86.4%//78.5%//79.4%<br>**Serious adverse events(w12) (P//22.5//75//225):** 7.9%//16.7%//13.8%//16.2%<br>**Dose reduction or temporary interruption due to adverse events (w12) (P//22.5//75//225):** 0%//3%//4.6%//2.9%<br>**Permanent discontinuation due to adverse events (w12) (P//22.5//75//225):** 4.8%//13.6%//12.3%//5.9%

SC: subcutaneous; w8: week 8; CDAI: Crohn's Disease Activity Index; P: placebo; 22.5: PF-00547659 22.5mg; 75: PF-00547659 75mg; 225: PF-00547659 225mg; w12: week 12; GMR: Geometric Mean Reduction; FCP: faecal calprotectin; hs-CRP: high-sensitivity C-reactive protein; *: increase; MS: median suppression; MAdCAM: mucosal addressin cell adhesion molecule

Table S8. Efficacy and safety results of **PF-00547659** (continuation)

Reference (Trial identification)	Design	Phase	Intervention group	Control group	Treatment period	Effectiveness
Hassan-Zahraee M et al. 2018 [56] OPERA (NCT01276509)	- Multicentric<br>- Randomized<br>- Placebo controlled<br>- Double-blind<br>- Parallel	2	**Age:** 18-75 years<br><br>-PF-00547659 22.5mg SC Weeks 0, 4 and 8 (n=66)<br><br>-PF-00547659 75mg SC Weeks 0, 4 and 8 (n=65)<br><br>-PF-00547659 225mg SC Weeks 0, 4 and 8 (n=68)	**Age:** 18- 75 years<br><br>-Placebo SC Weeks 0, 4 and 8 (n=63)	12 Weeks	**GMR of the serum concentration of soluble MAdCAM (w12/RB):**<br><br>-Placebo: 5.8%*<br>-PF-00547659 22.5mg: 87.1%<br>-PF-00547659 75mg: 95.4%<br>-PF-00547659 225mg: 97.7%
						**hs-CRP (w12/RB):**<br><br>-Placebo: 18.9/19.9 mg/l<br>-PF-00547659 22.5mg: 21.1/11.8 mg/l<br>-PF-00547659 75mg: 14.7/9.9 mg/l<br>-PF-00547659 225mg: 17.2/15.6 mg/l
						**FCP (w12/RB):**<br><br>-Placebo: 1797/1678 µg/g<br>-PF-00547659 22.5mg: 1705/987 µg/g<br>-PF-00547659 75mg: 1389/1066 µg/g<br>-PF-00547659 225mg: 1346/1769 µg/g
						**Increase in *CCR9* gene expression (w12):**<br><br>-Placebo: 1.03 times<br>-PF-00547659 22.5mg: 2.78 times<br>-PF-00547659 75mg: 2.8 times<br>-PF-00547659 225mg: 3.72 times

SC: subcutaneous; GMR: Geometric Mean Reduction; MAdCAM: mucosal addressin cell adhesion molecule; w12/RB: week 12 respect to the baseline; *: increase; hs-CRP: high-sensitivity C-reactive protein; FCP: faecal calprotectin; w12: week 12
